# Host specificity and horizontal gene transfer in the MIB-MIP immunoglobulin evasion system in *Mycoplasma gallisepticum*

**DOI:** 10.1186/s13567-026-01779-x

**Published:** 2026-08-11

**Authors:** Dilhani Ekanayake, Paola Vaz, Alistair R. Legione, Nadeeka K. Wawegama, Glenn F. Browning, Kelly A. Tivendale

**Affiliations:** Asia-Pacific Centre for Animal Health, Veterinary Preclinical Centre, Melbourne Veterinary School, Faculty of Science, The University of Melbourne, Parkville, VIC 3010 Australia

**Keywords:** *Mycoplasma gallisepticum*, MIB-MIP system, immunoglobulin-binding, immunoglobulin-cleavage, immune evasion, protease, virulence

## Abstract

*Mycoplasma gallisepticum* is a major poultry pathogen responsible for chronic respiratory disease and substantial global economic losses. Its ability to establish chronic infections reflects its effective immune evasion strategies, but the mechanisms underlying this remain poorly understood*.* Some pathogenic mammalian mycoplasmas use the Mycoplasma Immunoglobulin Binding-Protease (MIB-MIP) system to capture and cleave host immunoglobulins (Ig), but the functionality and host-specificity of this system in *M. gallisepticum* have not been examined. We aimed to functionally characterise all the MIB-MIP homologues in *M. gallisepticum* and examine their host specificity. Five putative MIB and five putative MIP genes of *M. gallisepticum* were cloned, expressed as recombinant GST-fusion proteins, and purified for functional analysis. Immunoglobulin-binding assays showed that all MIB proteins bound both avian and mammalian immunoglobulins, forming stable MIB-Ig complexes, with distinct binding capacities. In contrast, proteolytic assays revealed that only three of the five MIP proteins could cleave avian immunoglobulins, when complexed with any of the five MIBs, generating characteristic Ig heavy-chain fragments. Only one MIP protease showed detectable interaction with mammalian immunoglobulins, indicating strong host specificity of these MIPs and functional specialisation for avian immunoglobulin-cleavage. These results revealed that MIB and MIP proteins of *M. gallisepticum* are adapted to cleavage of avian immunoglobins, thereby interfering with antibody-mediated host immune responses. Bioinformatic analysis suggested that MIB-MIP homologues are widespread among avian mycoplasmas that share similar hosts, tissue tropisms and transmission patterns, and detected evidence of horizontal gene transfer and recombination, indicating that there have been MIB-MIP evolutionary adaptations among the avian mycoplasmas.

**Supplementary Information:**

The online version contains supplementary material available at 10.1186/s13567-026-01779-x.

## Introduction

Mycoplasmas are the simplest self-replicating prokaryotes. They are members of the class Mollicutes and have reductively evolved from Gram-positive eubacteria with a low G + C content [1, 2]. They have reduced genomes (0.5–1.5 Mbp), and as a result are obligate parasites, but have evolved to be successful colonizers of a broad array of hosts, including mammals, birds, reptiles, fish, arthropods and plants [1, 3]. Pathogenic mycoplasmas that cause chronic infections have preserved their genomic capacity to colonise and persist within their hosts by adopting sophisticated strategies to evade the immune responses of their hosts [4–6].

*Mycoplasma gallisepticum* is a poultry pathogen causing chronic respiratory disease (CRD) in chickens and infectious sinusitis in turkeys [7]. These diseases have major economic impacts on commercial poultry farming [7]. It has also been isolated from other avian hosts, including pheasants, bobwhite and Japanese quail, house finches, house sparrows, peafowl, chukar partridge, parrots and ducks [7–9]. In addition to establishing persistent respiratory tract infections, it can also disseminate systemically to distant niches [10], causing salpingitis [11], arthritis [12], keratoconjunctivitis [13] and encephalopathy [14]. It is also capable of surviving within recently vaccinated birds [7, 15], regardless of their humoral and cell-mediated immune responses. *M. gallisepticum* is known to use multiple immune evasion strategies, including antigenic and high-frequency phase variation, host immunomodulation and invasion of phagocytic and non-phagocytic host cells, including erythrocytes [4, 10, 16]. Antibody responses to mycoplasma infections and vaccination follow the typical pattern of an IgM response during the acute phase, progressing to high titres of IgG and IgA during the chronic phase [5, 17–19]. Studies on *Mycoplasma mycoides* subspecies *capri* have identified the Mycoplasma Immunoglobulin Binding (MIB) and Mycoplasma Immunoglobulin Protease (MIP) system, an immune evasion mechanism involving two cell surface expressed proteins [20]. In this system, the MIB protein functions as a high affinity IgG binding molecule, forming a MIB-IgG complex that recruits a MIP protease to cleave the bound IgG into its V_H_ and C_H1_ domains [20]. This system can also directly dissociate antigen–antibody complexes to release immunoglobulin-bound antigens [21]. By disrupting antibody structure and function, the MIB-MIP system can impair the host immune response and thus act as a virulence determinant in mycoplasmas [5, 20].

Genes encoding predicted homologues of the MIB-MIP system have been detected in the genomes of mycoplasmas infecting a number of animals, including *M. gallisepticum* [5, 20]. However, the function of these proteins in *M. gallisepticum* and their interactions with avian immunoglobulins have not been studied. The studies described here focussed on exploring the function of the products of these genes and their potential role in avian immunoglobulin evasion by *M. gallisepticum*, using recombinant GST-fusion proteins. Interactions with mammalian immunoglobulins were also examined to assess potential host-specific adaptations of the MIB-MIP system. From an evolutionary standpoint, this study also aimed to identify the distribution of MIB-MIP systems in other avian mycoplasmas and to evaluate their phylogenetic relationships with those of *M. gallisepticum*.

## Materials and methods

### Bioinformatic analysis

#### Predicting putative MIB-MIP homologues, and their structure and function

Putative MIB and MIP homologues in *M. gallisepticum* were identified using BLASTP and TBLASTN searches with the sequences of *M. mycoides* ssp. *capri (Mmc)* strain GM12 MIB (MMCAP2_0583; GenBank protein accession, ACU78312.1) and MIP (MMCAP2_0582; GenBank protein accession, ACU78687.1) proteins as the query sequences using the default settings. The nucleotide and protein sequences of putative MIB and MIP homologues identified in *M. gallisepticum* strain Ap3AS (GenBank: CP044624.1) were retrieved using Genious Prime 2023.0.1.

The protein sequences of the homologues were used to predict their cellular topology using DeepTMHMM [22], their functional domains using the Conserved Domain Search [23, 24] and their putative function and structure using Phyre^2^ [25]. HHpred analysis in the MPI Bioinformatics toolkit was used for remote homology detection using the PDB database, as updated on the 8^th^ May 2025, using the default settings [26, 27]. ProtParam (Expasy) was used to determine the amino acid sequence length and molecular mass (kDa) of the proteins.

A BLASTX search was performed using the Mold, Protozoan, and Coelenterate Mitochondrial Code and the Mycoplasma/Spiroplasma genetic code with default settings to identify the putative MIB and MIP homologues in different strains of *M. gallisepticum* and in other avian mycoplasma species. For these searches the nucleotide sequences of the MIB and MIP homologues of *M. gallisepticum* strain Ap3AS were used as the query sequences. The complete genome sequences of *M. gallisepticum* strains R_low_ (GenBank: NC_004829.2), R_high_ (GenBank: NC_017502.1), S6 (GenBank: CP006916.3), F (GenBank: NC_017503.1), and ts-11 (GenBank: NZ_CP044225.1), and other avian mycoplasmas, including *M. synoviae* strain 86079/7NS (GenBank: NZ_CP029258.1), *M. imitans* ATCC51306 (GenBank: NZ_KI912416.1), *M. bradburyae* strain T158 (GenBank: NZ_CP101414.1), *M. tullyi* strain 56A97T (GenBank: NZ_CP059674.1), *M. gallinarum* strain DSM19816 (GenBank: NZ_JHZE01000008.1), *M. meleagridis* strain NCTC10153 (GenBank: NZ_LR215042.1), *M. pullorum* strain B359_6 (GenBank: NZ_CP017813.1), *M. gallinaceum* strain NCTC10183 (GenBank: NZ_LR214950.1), *M. iners* strain ATCC19705 (GenBank: GCF_000701805.1), *M. anatis* strain NCTC10156 (GenBank: CP030141.1), *M. anseris* strain ATCC49234 (GenBank: CP030140.1), *M. cloacale* strain NCTC10199 (GenBank: CP030103.1), *M. gallopavonis* strain NCTC10186 (GenBank: LR215032.1) and *M. glycophilum* strain NCTC10194 (GenBank: LR215024.1), were retrieved from the NCBI database [28] on the 23rd June 2025 and used to extract the coding sequences of putative MIB and MIP homologues and their associated gene regions.

The pairwise identities of the MIB-MIP associated gene clusters and individual homologues of *M. gallisepticum* and other avian mycoplasma species were retrieved from the distance matrix table obtained after aligning their extracted coding sequences with Multiple Alignment with Fast Fourier Transformation (MAFFT) [29, 30] version 7.490 in Genious Prime version 2023.0.1.

The percentage amino acid sequence similarities and identities of the MIB and MIP homologues within *M. gallisepticum* strain Ap3AS, and in other strains of *M. gallisepticum* and other avian mycoplasma species were obtained using the pairwise Needleman-Wunsch Global Alignment of protein sequences using the default settings. Pseudogenes were excluded from these comparisons.

#### Alignment of protein sequences

Protein sequence alignments were generated using the EMBL-EBI Clustal Omega multiple sequence alignment program using the default parameters [31]. The alignment was then processed using ESPript 3.0 to generate a graphical alignment [32].

The protein sequences of the MIP homologues in *M. mycoides* ssp. *capri* strain GM12 (GenBank: NZ_CP001668.1), MMCAP2_0582 (GenBank: ACU78687.1), MMCAP2_0584 (GenBank: ACU78878.1), MMCAP2_0586 (GenBank: ACU78388.1), and MMCAP2_0588 (GenBank: ACU78592.1), were aligned and compared with the MIP homologues of *M. gallisepticum* strain Ap3AS. To identify the probable cleavage site in the immunoglobulin heavy chains, the available amino acid sequences of V_H_ domains of immunoglobulin heavy chains of chicken IgY (GenBank: BDI00299.1), duck IgY (GenBank: PIR: B46529), goat IgG (GenBank: ABY65927.1), sheep IgG (GenBank: AAD52606.1), rat IgG (GenBank: AAA41393.1), rabbit IgG (GenBank: AAD14233.1), horse IgG (GenBank: AAG01011.1) and pig IgG (GenBank: NP_998993.1) were retrieved from the NCBI database [28] on the 20^th^ June 2025.

Protein sequences of the putative MIB and MIP homologues identified in avian mycoplasmas were aligned with the MIB and MIP homologues in *M. gallisepticum* Ap3AS. Individual protein accession numbers are shown in Additional file 1.

#### Analysis for gene recombination, horizontal gene transfer and gene rearrangement

Phylogenetic inferences for the MIB and MIP homologues were analysed using split decomposition. The nucleotide sequences of the MIB and MIP homologues were aligned using MAFFT version 7.490 [29, 30] in Genious Prime, and the FASTA file was imported into SplitsTree CE 6.0.0 [33] to generate the split decomposition network. The MIB alignment comprised 66 taxa with a total length of 4789 bp, while the MIP alignment comprised 55 taxa with a total length of 4,165 bp. The P Distance method [34] was used with the default options to generate 66 × 66 and 55 × 55 distance matrixes for the MIB and MIP datasets, respectively. The Neighbour Net method [35, 36] was used with the default options to obtain 207 splits for MIB homologues and 175 splits for MIP homologues and to create cyclic networks. The Show-Splits method was used with the default options to obtain a Split Network visualization.

The PHI test method [37] was used to evaluate the significance of the recombination networks within each of the sets of homologues. The clusters closely related to the *M. gallisepticum* homologues were separately evaluated for recombination. Recombination was further evaluated using RDP4.101 [38], using the RDP, GNECONV, BootScan, Maximum Chi Square (MaxChi), Chimasera, SiScan and 3Seq recombination detection methods and the default settings, with a highest acceptable *p*-value of 0.05. Recombination events confirmed by both the split decomposition network and RDP4 analyses were considered significant.

Regions of the genome encoding the MIB-MIP systems and the genes immediately adjacent to them were extracted from the complete genomes of the different *M. gallisepticum* strains and the other avian mycoplasma species. Their nucleotide sequences were aligned with those of *M. gallisepticum* strain Ap3AS, using the progressive Mauve algorithm [39] in Genious Prime, using the default settings, to identify homologous sites and gene rearrangements.

### Bacterial strains and media

High-efficiency NEB 5-alpha competent *E. coli* cells (NEB, USA) were used for plasmid maintenance and BL21 (DE3) competent *E. coli* cells (NEB) were used for expression of the recombinant peptides.

Plasmid-transformed *E. coli* cells were cultured at 37 °C in Luria–Bertani (LB) broth (1% w/v tryptone (Oxoid, UK), 0.5% w/v yeast extract (Oxoid), 0.5% w/v NaCl (Ajax Finechem, Australia) for 18 h with shaking (200 rpm) or on LB agar (1% bacteriological agar (Oxoid) in LB broth) containing ampicillin at 50 µg.mL^−1^ (Sigma-Aldrich, USA).

*E. coli* cultures in LB broth were stored in 40% glycerol (Ajax FineChem) at −80 °C. *E. coli* cell pellets were frozen at −20 °C for short-term storage and at −80 °C for long-term storage.

### Cloning

Genious Prime version 2023.0.1 was used to extract the coding sequences (CDS) from the Ap3AS genome and to identify the TGA codons that would lead to premature truncation of translation of the proteins in *E. coli*. Signal peptides and their cleavage sites were predicted using the SignalP-6.0 server. Nucleotide sequences of proteins with predicted signal peptides were modified to produce insert sequences that could be cloned into the expression vector pGEX-4T-1. These sequences introduced a BamHI site at the 5′ end, immediately after the cysteine (C) codon of the predicted signal peptide cleavage site. TGA codons were altered to TGG to enable full-length expression of the gene in *E. coli*. The stop codon encoded by the gene was followed by two additional termination codons incorporating a SalI restriction site at the 3′ end.

Modified gene sequences were synthesised and provided in the pUC57 vector as 4 µg of lyophilized DNA (GenScript, USA). The DNA was reconstituted to 200 ng.µL^−1^ and digested with BamHI-HF (NEB) and SalI-HF (NEB) according to the manufacturer’s instructions. Following digestion, pUC57 and the insert DNA fragments were separated by 1% agarose gel electrophoresis. Insert DNA fragments were excised from the gel and purified using the Monarch DNA Gel Extraction Kit (NEB) according to the manufacturer's instructions. Insert DNA fragments were then ligated into BamHI-HF and SalI-HF digested pGEX-4T-1 (Cytiva, USA) using T4 DNA Ligase (NEB) according to the manufacturer’s instructions.

High-efficiency NEB 5-alpha competent *E. coli* cells were transformed with 1 µL of ligation mix according to the manufacturer’s instructions. A single ampicillin-resistant *E. coli* colony was selected and cultured in LB broth containing ampicillin at 50 µg.mL^−1^. Plasmid DNA was extracted using the Wizard Plus SV Minipreps DNA Purification System (Promega, USA) according to the manufacturer's instructions. Purified plasmid DNA was stored at −20 °C. Clones containing an insert of the correct size in the pGEX-4T-1 expression vector were identified by restriction endonuclease digestion of plasmid DNA with BamHI-HF and SalI-HF, followed by 1% agarose gel electrophoresis. Sequences of the inserts in the pGEX-4T-1 transformants were confirmed by Sanger dideoxy sequencing of extracted plasmid DNA using the pGEX_F (forward 5’-CCAGCAAGTATATAGCATGGCC-3’) and pGEX_R (reverse 5’-CTCCGGGAGCTGCATGTG-3’) primers, along with internal primers where required (Additional file 2).

BL21 (DE3) competent *E. coli* cells were transformed with 1 µL of purified plasmid DNA according to the manufacturer’s instructions. A single ampicillin-resistant colony was selected and cultured in LB broth containing ampicillin at 50 µg.mL^−1^. Successfully transformed clones were confirmed to contain the desired recombinant plasmids by extracting the plasmid DNA, digesting it with BamHI-HF and SalI-HF, and separating the DNA fragment by 1% agarose gel electrophoresis as described above.

### Protein expression and purification

Overnight cultures of transformed BL21 (DE3) *E. coli* cells in LB broth were diluted 1:100 and incubated at 37 °C with shaking at 200 rpm until they reached an A_600_ of 0.6 ± 0.1. The expression of the glutathione-S-transferase (GST) tagged fusion proteins in BL21 (DE3) *E. coli* cells was induced by adding isopropyl-beta-D-thiogalactopyranoside (IPTG) (Sigma-Aldrich) to a final concentration of 1 mM, then incubating the culture at 37 °C with shaking for a further 3–4 h. The *E. coli* cells were pelleted, resuspended in 1 × phosphate-buffered saline (PBS), lysed overnight at 4 °C with lysozyme (1 mg.mL^−1^) and sonicated (20–25 short bursts of 15 s each, on ice). Triton X-100 was added to a final concentration of 0.2% and the lysate then incubated for 30 min at 4 °C. The recombinant proteins were purified from the soluble protein fraction by affinity chromatography on a glutathione Sepharose column (Cytiva) at 4 °C according to the manufacturer’s instructions. GST-tagged recombinant peptides were eluted from the column using glutathione elution buffer (10 mM glutathione in 50 mM Tris–HCl pH 8.0). Eluted proteins were dialysed against PBS at 4 °C and stored at −80 °C. GST alone expressed from pGEX-4T-1 in BL21 (DE3) competent *E. coli* cells was purified using the same method to serve as a negative control.

Protein concentrations were measured using the Pierce BCA Protein Assay (Thermo Fisher Scientific, USA) according to the manufacturer’s instructions. The expected molecular masses of the recombinant proteins were calculated from the sequence of the insert using ProtParam (Expasy), with 26 kDa was added to account for the molecular mass of the GST tag.

### SDS-PAGE analysis and western blotting

Protein samples (5 µL) were mixed with 5 µL of 2 × reducing lysis buffer (62.5 mM Tris–HCl, 25% v/v glycerol, 2% w/v SDS and 0.01% w/v bromophenol blue, 5% v/v β-mercaptoethanol), incubated at 100 °C for 10 min, and the proteins separated by SDS-PAGE in 10% polyacrylamide gels (TGX Stain-Free FastCast Acrylamide Kit, Bio-Rad) at 180 V for 40 min, alongside a protein marker (Precision Plus Protein Standards-Unstained, Bio-Rad). For non-reducing gels, samples were prepared without β-mercaptoethanol or incubation at 100 °C and separated in 4–20% gradient gels (Mimi-Protein TGX Stain-Free, Acrylamide Kit, Bio-Rad) using the same electrophoresis conditions.

Separated proteins were transferred onto a PVDF membrane (Bio-Rad) using the Trans-Blot Turbo transfer system (Bio-Rad). The membrane was saturated overnight at 4 °C in a blocking solution of 5% (w/v) skim milk powder in 0.1% (v/v) PBST (1 × PBS, 0.1% (v/v) Tween-20). The membrane was washed three times for 5 min each in 30 mL of 0.1% (v/v) PBST.

Each recombinant protein was confirmed to contain the GST fusion partner by probing western blots with goat anti-GST antibody (GE Healthcare, UK) and polyclonal rabbit anti-goat HRP-conjugated antibody (Dako, USA), both diluted 1:2000 in 1% (w/v) skim milk in 0.1% (v/v) PBST. Membranes were incubated with the anti-GST antibody for 1 h, washed three times for 5 min each with 0.1% (v/v) PBST, then incubated with the anti-goat conjugate for 1 h and washed three times for 5 min each with 0.1% (v/v) PBST. Bound antibody was detected using the Clarity Western ECL Substrate (Bio-Rad) according to the manufacturer’s instructions and the blots imaged using the ChemiDoc MP system (Bio-Rad).

Chicken IgY heavy and light chains were detected using goat anti-chicken IgY (H + L) HRP-conjugated antibody (Bio-Rad), while IgY heavy chain Fc fragments were detected using goat anti-chicken IgY Fc HRP-conjugated antibody (Bio-Rad). Washed membranes were incubated with the antibody diluted 1:2000 in 1% (w/v) skim milk powder in 0.1% (v/v) PBST for 1 h. Chicken IgM was detected by incubating the washed membranes for 1 h with goat anti-chicken IgM antibody (Bio-Rad) diluted 1:1500, followed by washing and incubation for 1 h with polyclonal rabbit anti-goat HRP-conjugated secondary antibody (Dako) diluted 1:2000. Chicken IgA was detected by incubating the washed membranes for 90 min with goat anti-chicken IgA HRP-conjugated antibody (Bio-Rad) diluted 1:600. Unbound antibodies were removed by washing, and the bound antibodies detected, as described above.

### Animal sera

The animal sera used in this study were obtained from various members of the Asia–Pacific Centre for Animal Health (APCAH) at The University of Melbourne (chicken serum from Dr. Pollob Shil, turkey, pheasant and pig sera from Anna Kanci Condello, goat serum from Assoc. Prof. Simon Firestone, sheep serum from Dr. Nadeeka Wawegama, rat and rabbit sera from Assoc. Prof. Carol Hartley, horse serum from Kim Carey, and tammar wallaby serum from Dr Paola Vaz). The duck serum was obtained from Prof. Marcel Klaassen from the School of Life and Environmental Sciences, Deakin University.

### Immunoglobulin binding assay

The capacity of the MIB proteins to bind immunoglobulin was assessed by incubating the recombinant GST-MIB proteins with chicken serum. Chicken serum was diluted 1:10 in 1 × PBS and filtered using a 0.45 µm Millex-HV PVDF low protein binding filter (Sigma-Aldrich). The diluted serum was mixed with 10 mM purified GST-MIB protein. The mixture was incubated overnight at 4 °C and then at room temperature for 4 h with gentle agitation to enhance binding. The same protein concentrations were used for all experiments so that the binding capacities of the different GST-MIB proteins could be compared. The MIB protein-immunoglobulin complexes were purified using GST SpinTrap columns (Cytiva). Columns were equilibrated with 600 µL of binding buffer (140 mM NaCl, 2.4 mM KCl, 10 mM Na_2_HPO_4_, 1.8 mM KH_2_PO_4_, pH 7.3), and the GST-MIB and serum mixture was added and the column then incubated for 1 h at room temperature to allow optimal binding. Columns were washed three times with 600 µL of binding buffer and the bound complexes were eluted by adding 200 µL of elution buffer (50 mM Tris–HCl, 10 mM reduced glutathione, pH 8.0) and incubating the column for 30 min at room temperature before recovering the eluate. The eluted protein products were separated by SDS-PAGE in 10% polyacrylamide gels. Purified recombinant GST-MIB proteins alone and diluted chicken serum alone were used as controls. Binding of GST-MIB proteins with chicken IgY, IgM and IgA were confirmed by western blotting, as described above. Binding assays with sera from other avian species and mammals, to assess the capacity of *M. gallisepticum* MIB proteins to bind immunoglobulins from other animal species, were performed using the same method. Binding assays were also performed using purified chicken IgY (Bio-Rad) at a final concentration of 0.5 mg.mL^−1^.

### Immunoglobulin cleavage assay

The protease activity of the recombinant GST-MIP proteins was evaluated in vitro by incubating the eluates from the binding assays with 6 mM purified GST-MIP protein at 37 °C for 6 h. Cleavage capacities of the MIP proteins were compared by using the same concentrations of eluates and GST-MIP proteins for all assays. Following incubation, the protein products were separated by SDS-PAGE in 10% polyacrylamide gels, and immunoblotting using goat anti-chicken IgY Fc HRP-conjugated antibody was used to confirm cleavage of the IgY heavy chain. Cleavage of the chicken IgM and IgA heavy chains was confirmed separately using goat anti-chicken IgM and goat anti-chicken IgA antibodies. Controls included purified recombinant GST-MIP proteins alone and diluted chicken serum alone. The assays were also performed using MIB-bound immunoglobulins in eluates from the binding assays using sera from other animal species.

The direct cleavage capacity of recombinant GST-MIP proteins was assessed by incubating purified GST-MIP proteins with purified chicken IgY (Bio-Rad) at final concentrations of 2 mM MIP and 1 mM IgY, and then incubating the mixture for 6 h at 37 °C to assess the ability of GST-MIP proteins to cleave unbound IgY directly. Protein products were separated by SDS-PAGE and western blots were probed with goat anti-chicken IgY Fc HRP conjugated antibody to detect cleavage of the IgY heavy chains.

## Results

### *M. gallisepticum* encodes multiple putative MIB-MIP homologues arranged in separate gene clusters

The genome of *M. gallisepticum* strain Ap3AS (GenBank: CP044624.1) was predicted to contain five putative MIB homologues: *F9C22_03940*, *F9C22_03935*, *F9C22_03930*, *F9C22_03910* and *F9C22_03850* (GenBank protein accessions UQZ95866.1, UQZ95865.1, UQZ95864.1, UQZ95861.1 and UQZ95854.1, respectively), with predicted molecular masses between 75 and 78 kDa and amino acid lengths from 672 to 703 residues (Table 1). DeepTMHMM analysis predicted these proteins to be surface-expressed membrane proteins, each possessing an N-terminal signal peptide sequence of 21–29 amino acids. Conserved Domain Searches predicted a Mycoplasma- and Ureaplasma-specific immunoglobulin blocking virulence protein domain covering the majority of their sequence. HHpred and Phyre^2^ analyses indicated that these proteins shared high structural similarity with the functional MIB proteins of *M. mycoides* ssp. *capri*, with additional similarity to the antibody binding Protein M (MG281) of the human pathogen *M. genitalium*. *M. gallisepticum* also carries a pseudogene, *F9C22_03915*, that was identified as a frameshifted putative MIB homologue with multiple stop codons.

**Table 1:** Features of putative MIB and MIP gene homologues in *M. gallisepticum* strain Ap3AS.

	Gene locus tag/ORF	Genome region (bp)^a^	Gene length^a^ (bp)	Protein length^b^ (aa)	Protein size^b^ (kDa)	Number of TGA codons^a^
MIB homologues	*F9C22_03940*	985,413–987,518	2,106	701	78	8
	*F9C22_03935*	983,124–985,235	2,112	703	78	7
	*F9C22_03930*	980,974–982,998	2,025	674	76	8
	*F9C22_03910*	973,197–975,272	2,076	691	77	4
	*F9C22_03850*	959,363–961,381	2,019	672	75	10
MIP homologues	*F9C22_03920*	977,485–979,860	2,376	791	89	9
	*F9C22_03865*	962,256–964,646	2,391	796	89	9
	*F9C22_03660*	898,248–900,287	2,040	679	76	7
	*F9C22_03655*	896,322–898,157	1,836	611	68	8
	*F9C22_00075*	15,030–17,282	2,253	750	83	11

^a^Genious Prime version 2023.0.1 was used to retrieve these parameters

^b^ProtParam (Expasy) was used to compute these parameters

Four putative MIP homologues*: F9C22_03920, F9C22_03865, F9C22_03660,* and *F9C22_03655* (GenBank protein accessions UQZ95862.1, UQZ95855.1, UQZ95823.1, UQZ95822.1, respectively) and an additional DUF31-containing protein *F9C22_00075* (GenBank protein accession UQZ95205.1) were identified in *M. gallisepticum*. These proteins were predicted to be surface-expressed lipoproteins (611–796 amino acids; 68–89 kDa) (Table 1) containing a mycoplasma-specific DUF31 serine protease domain and a conserved catalytic site (S_693_ in *F9C22_03920*, S_695_ in *F9C22_03865*, S_583_ in *F9C22_03660*, S_528_ in *F9C22_03655*, and S_644_ in *F9C22_00075*). HHpred analysis predicted a lipoprotein motif and structural similarity to the antibody binding protein-protease complex of *M. mycoides* ssp. *capri*. These putative MIP proteins only shared 31–43% amino acid sequence similarity (18–28% identity) with the functional MIP proteins in *M. mycoides* ssp. *capri*, with higher amino acid sequence conservation towards the C-terminal end near the catalytic site (Additional files 3 and 4).

**Figure 1:**
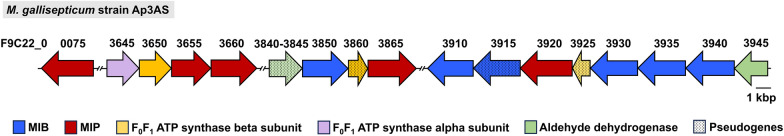
**Distribution of putative MIB-MIP homologues in the *M. gallisepticum* Ap3AS genome**. The putative MIB (blue arrowhead) and MIP (red arrowhead) homologues are arranged in separate gene clusters with the closely associated putative F_0_F_1_ ATP synthase beta (yellow arrowheads) and alpha (purple arrowhead) subunit genes and pseudogenes. Frameshifted or truncated pseudogenes are indicated by vertical dashed hatching. Green arrowheads indicate the putative aldehyde dehydrogenase genes and pseudogenes.

The putative MIB-MIP homologues of *M. gallisepticum* were arranged in three distinct gene clusters at separate genomic loci (Figure 1). Two clusters contained paired MIB and MIP genes adjacent to truncated pseudogenes annotated as putative F_0_F_1_ ATP synthase subunits, while a third locus contained two adjacent MIP homologues downstream of putative F_0_F_1_ ATP synthase alpha and beta subunit genes (Figure 1). The putative MIP homologue *F9C22_00075* was located independently at a separate gene locus (Figure 1). Multiple genome alignments revealed a conserved MIB-MIP gene arrangement across other *M. gallisepticum* strains, including R_low_, R_high_, S6, F and ts-11) (Additional files 1 and 5), with pairwise nucleotide identities ranging from 95 to 99% and over 90% amino acid sequence similarity and identity (Additional files 6 and 7). Notably, some homologues, *MGA_RS03960* (R_low_), *MGAH_RS03945* (R_high_), and *F6J64_RS03900* and *F6J64_RS03880* (ts-11), were predicted to be pseudogenes due to frameshift mutations leading to premature stop codons (Additional files 1 and 5).

### The putative MIB-MIP paralogues in *M. gallisepticum* show considerable heterogeneity

The MIB and MIP paralogues in *M. gallisepticum* strain Ap3AS exhibited considerable nucleotide and protein sequence diversity. The MIB paralogues shared 34–74% pairwise nucleotide identity (Additional file 8), and 45–82% amino acid similarity (28–71% identity) (Additional file 9), with higher amino acid sequence conservation at their N-terminus (Additional file 10). The most conserved putative MIB proteins were *F9C22_03930* and *F9C22_03850* (82% similarity, 71% identity), followed by the homologues *F9C22_03940*, *F9C22_03935*, and *F9C22_03910* (62–67% similarity, 47–54% identity) (Additional file 9).

The MIP paralogues had 26–79% pairwise nucleotide identity (Additional file 11), while their amino acid sequences had 29–84% similarity (17–72% identity) (Additional file 12), with a highly conserved C-terminus that included the catalytic serine site (Additional file 13). Among these, the proteins encoded by *F9C22_03920* and *F9C22_03865* shared the highest amino acid similarity of 84% (72% identity) (Additional file 12), even though they were located in separate gene clusters.

### Expression of recombinant MIB and MIP proteins of *M. gallisepticum*

**Figure 2:**
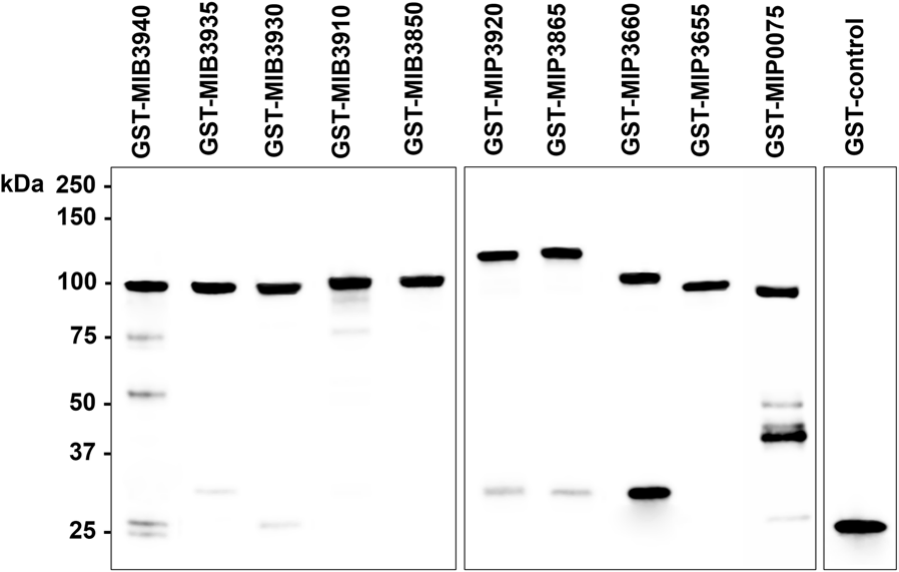
**Western blots of the purified recombinant GST-fusion putative MIB and MIP proteins of *M. gallisepticum***. The purified GST-fused recombinant MIB and MIP proteins had their expected apparent molecular weights relative to the Precision Plus Protein Standards: GST-MIB3940 (100 kDa), GST-MIB3935 (101 kDa), GST-MIB3930 (97 kDa), GST-MIB3910 (99 kDa), GST-MIB3850 (97 kDa), GST-MIP3920 (112 kDa), GST-MIP3865 (112 kDa), GST-MIP3660 (99 kDa), GST-MIP3655 (92 kDa), GST-MIP0075 (107 kDa). Purified GST (26 kDa) was included as a control.

To functionally characterise the genes encoding the putative MIB and MIP proteins, recombinant GST fusion proteins were expressed in *E. coli*. The recombinant GST-MIB proteins GST-MIB3940, GST-MIB3935, GST-MIB3930, GST-MIB3910, and GST-MIB3850 had apparent molecular weights of 100 kDa, 101 kDa, 97 kDa, 99 kDa, and 97 kDa, respectively, while the recombinant GST-MIP proteins GST-MIP3920, GST-MIP3865, GST-MIP3660, GST-03655 and GST-MIP0075 had apparent molecular weights of 112 kDa, 112 kDa, 99 kDa, 92 kDa and 107 kDa, respectively (Figure 2).

### All five MIB proteins of *M. gallisepticum* bind avian immunoglobulins

**Figure 3:**
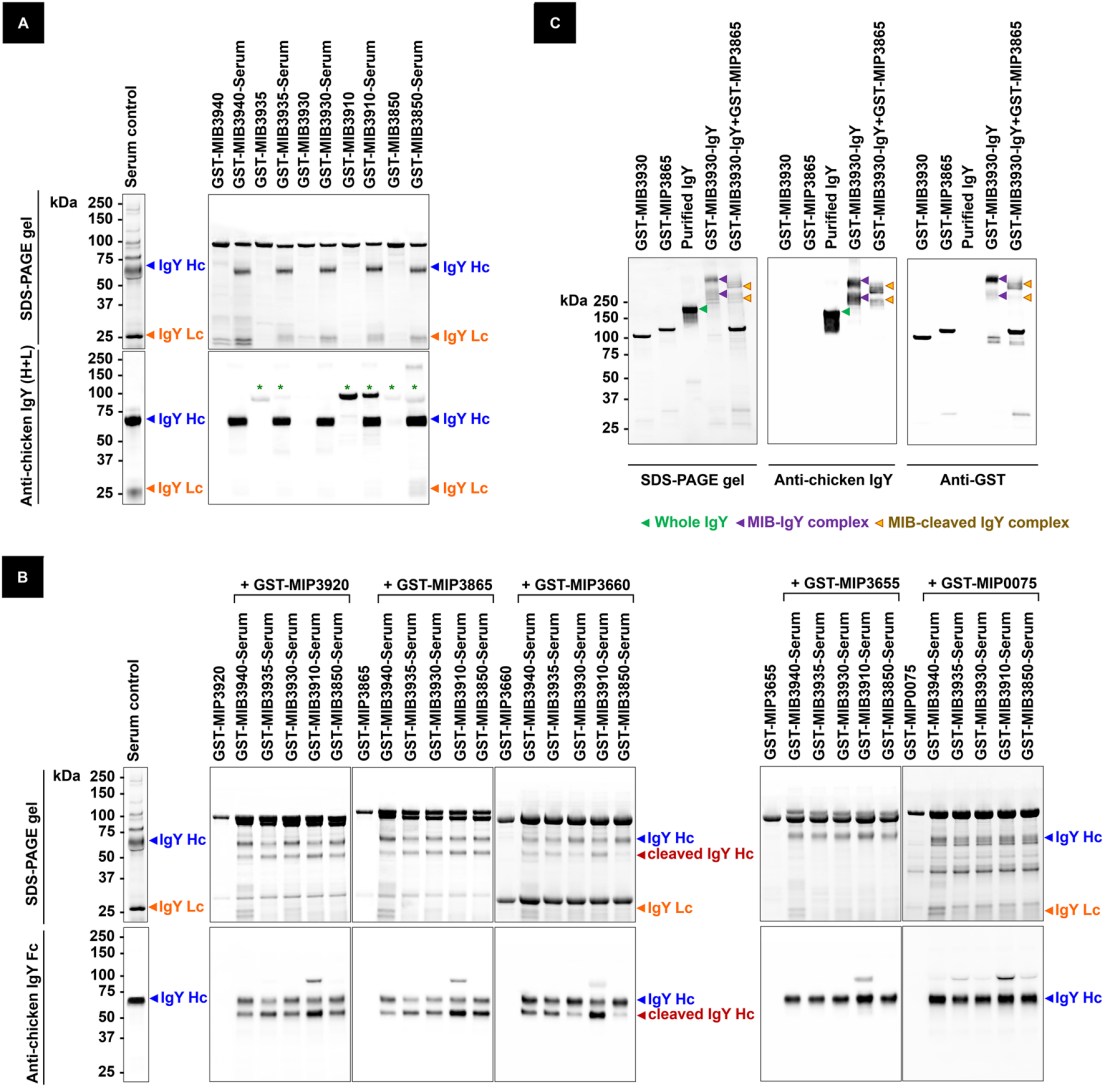
**In vitro interaction of putative MIB-MIP proteins of *M. gallisepticum* with chicken IgY**. **A** IgY binding capacity of the putative recombinant GST-MIB proteins. The GST-MIB-Serum lanes contain purified complexes of GST-MIB proteins with IgY (GST-MIB3940, GST-MIB3935, GST-MIB3930, GST- MIB3910 and GST-MIB3850) after incubation with diluted chicken serum. Complexes were resolved by SDS-PAGE and probed with goat anti-chicken IgY (H + L) HRP-conjugated antibody. GST-MIB proteins alone and diluted chicken serum (serum control) were included as controls. Blue arrowheads indicate the IgY heavy chain (~65 kDa); orange arrowheads indicate the IgY light chain (~25 kDa); green asterisks (*) indicate binding of the conjugated goat IgG to GST-MIB proteins. **B** Proteolytic cleavage of MIB-bound IgY by MIP proteases. Eluates with GST-MIB bound IgY (GST-MIB-Serum lanes) were incubated with purified putative recombinant GST-MIP proteins (GST-MIP3920, GST-MIP3865, GST-MIP3660, GST-MIP3655 and GST-MIP0075). Products were resolved by SDS-PAGE and probed with anti-chicken IgY Fc HRP conjugated antibody. GST-MIP proteins alone and diluted chicken serum (serum control) were included as controls. Blue arrowheads indicate the intact IgY heavy chain (~65 kDa), red arrowheads cleaved IgY heavy chain (~50 kDa) and orange arrowheads IgY light chain (~25 kDa). **C** MIB-MIP interaction with purified chicken IgY under non-reducing conditions. GST-MIB proteins (only GST-MIB 3930 is shown) were incubated with purified chicken IgY. Purified bound complexes (GST-MIB-IgY) were incubated with GST-MIP proteases (only GST-MIP3865 is shown), and the resultant protein products were separated by non-reducing SDS-PAGE and probed with goat anti-chicken IgY Fc HRP-conjugated antibody and goat anti-GST antibody. GST-MIB3930, GST-MIP3865, and purified chicken IgY alone were included as controls. Green arrowheads indicate the intact IgY (~180 kDa), purple arrowheads indicate monomeric and dimeric MIB-IgY complexes (>250 kDa), and yellow arrowheads indicate monomeric (~250 kDa) and dimeric (>250 kDa) MIB-cleaved IgY complexes.

**Figure 4:**
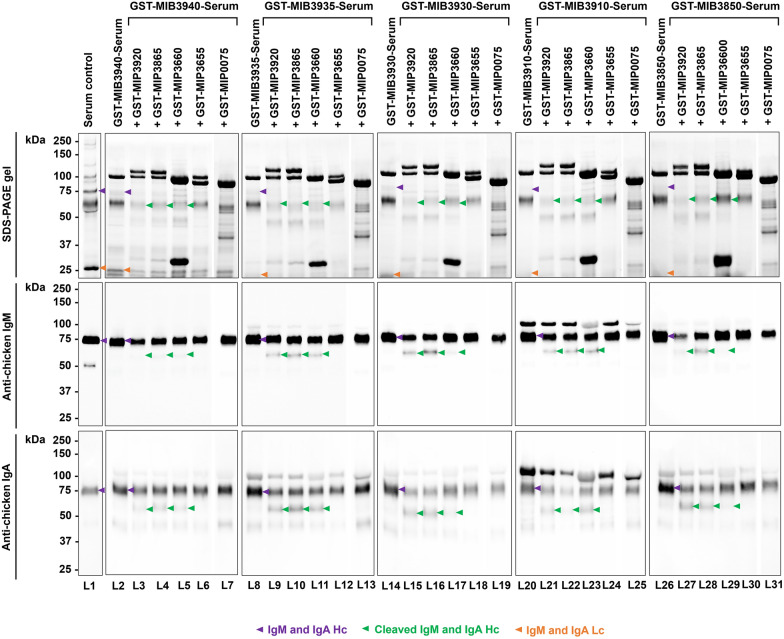
**Binding and cleavage of chicken IgM and IgA by the putative MIB-MIP proteins in *M. gallisepticum***. Eluates of the binding assay for the five GST-MIB proteins (indicated by lanes: L2, L8, L14, L20 and L26) were incubated with each of the five GST-MIP proteins (L3–L7, L9–L13, L15–L19, L21–L25 and L27–L31), resolved by SDS-PAGE and probed with goat anti-chicken IgA or goat anti-chicken IgM antibodies. Purple arrowheads indicate intact heavy chains (~75 kDa), green arrowheads cleaved heavy chains (~60 kDa), and orange arrowheads light chains (~25 kDa).

The immunoglobulin binding capacity of the recombinant GST-MIB proteins GST-MIB3940, GST-MIB3935, GST-MIB3930, GST-MIB3910, and GST-MIB3850 was assessed by incubating each of them with diluted chicken serum and then purifying the protein complexes using GST SpinTrap columns. SDS-PAGE analysis of the purified complexes revealed two protein bands of ~ 65 kDa and ~25 kDa, along with the ~100 kDa band corresponding to the GST-MIB protein (Figure 3A) for all five putative MIB proteins. Western blotting confirmed these to be the ~65 kDa heavy chain the ~25 kDa light chain of IgY and the recombinant proteins (Figure 3A), indicating that all five MIB proteins were capable of binding the entire 180 kDa IgY in chicken serum. Some of the MIB proteins cross-reacted with goat serum immunoglobulins and displayed differing binding capacities for immunoglobulins (Figure 3A). A faint protein band of ~ 75 kDa just above the IgY heavy chain of ~ 65 kDa was observed for all five GST-MIB proteins following incubation with chicken serum (Figure 4). This band was found to contain the IgM and IgA heavy chains by probing western blots with antibodies against IgM or IgA (Figure 4). Binding assays were also performed with turkey, pheasant and duck sera, revealing that the MIB proteins were capable of binding IgY, IgM and IgA from other avian hosts of *M. gallisepticum* (Additional file 14).

### *M. gallisepticum* has only three functional MIP proteins that cleave MIB-bound avian immunoglobulins

The protease activity of the recombinant GST-MIP proteins GST-MIP3920, GST-MIP3865, GST-MIP3660, GST-MIP3655 and GST-MIP0075 was evaluated by incubating the proteins individually with the eluates from the MIB binding assay. SDS-PAGE and western blotting showed that three of the putative MIP proteins — GST-MIP3920, GST-MIP3865 and GST-MIP3660 — cleaved the ~65 kDa IgY heavy chain of MIB-bound IgY, resulting in a cleaved heavy chain of ~50 kDa with an intact Fc portion (Figure 3B). Their cleavage capacity varied depending on the MIB variant, as reflected by differences in band intensity (Figure 3B). No IgY cleavage was detected with GST-MIP3655 or GST-MIP0075 (Figure 3B). All five GST-MIP proteins were also screened for proteolytic activity against IgM and IgA (Figure 4). Although the cleaved heavy chain was not evident following SDS-PAGE, western blotting confirmed that all MIP proteins, except GST-MIP3655 and GST-MIP0075, cleaved both IgM and IgA heavy chains to yield a ~60 kDa protein, which was otherwise obscured by the uncleaved IgY heavy chain (Figure 4). Similar cleavage patterns were observed with turkey, pheasant and duck sera (Additional file 14). Recombinant GST-MIP proteases did not cleave purified chicken IgY in the absence of the MIB proteins, with the ~65 kDa IgY heavy chain remaining intact in SDS-PAGE gels and western blots, indicating that MIP proteins do not directly interact with unbound IgY (Additional file 15).

### Chicken IgY remains tightly bound to MIB proteins following proteolytic cleavage by MIP proteases

Two high molecular weight bands above 250 kDa (~280 kDa and ~380 kDa) were observed after SDS-PAGE under non-reducing conditions of the eluates from the binding assay using purified chicken IgY (Figure 3C). Western blotting with anti-IgY and anti-GST antibodies confirmed that both bands contained IgY and MIB, consistent with the formation of stable MIB-IgY complexes comprising one IgY molecule (~180 kDa) bound to either one MIB (~280 kDa) or two MIB (~380 kDa) proteins (Figure 3C). Following proteolytic cleavage by the MIP protease, additional bands appeared just below the MIB-IgY complexes, one slightly above 250 kDa and another at 250 kDa, indicating that the cleaved IgY fragment remained tightly bound by the MIB proteins (Figure 3C).

### The MIB-MIP proteins of *M. gallisepticum* vary in their interactions with mammalian immunoglobulins

**Figure 5:**
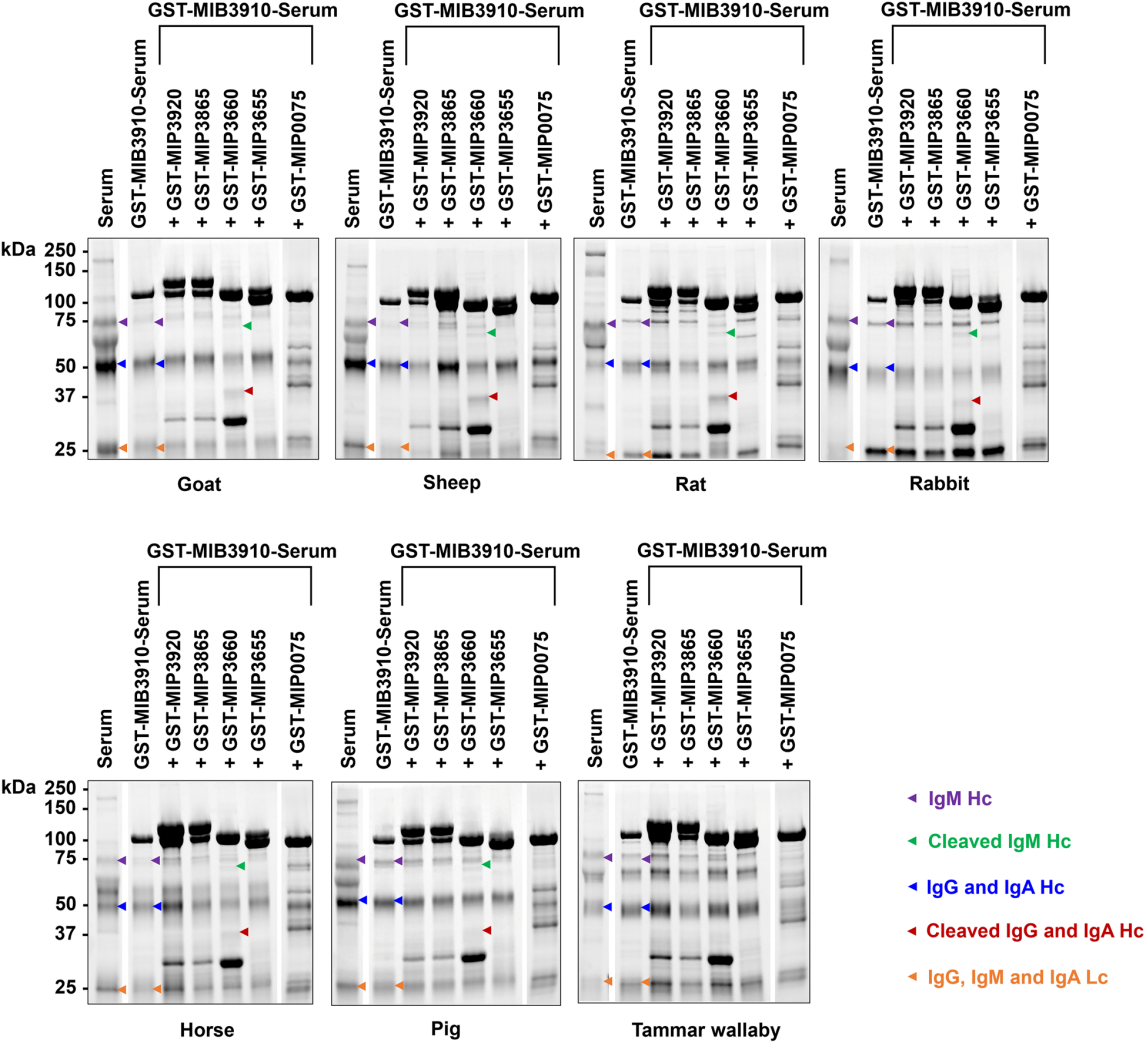
**Interaction of putative MIB-MIP proteins of *M. gallisepticum* with mammalian immunoglobulins.** Purified recombinant GST-MIB proteins (only GST-MIB3910 is shown as representative of the observations for all five MIB proteins) were incubated with diluted goat, sheep, rat, rabbit, horse, pig and tammar wallaby serum. Affinity purified MIB-immunoglobulin complexes were then incubated with purified recombinant GST-MIP proteins (GST-MIP3920, GST-MIP3865, GST-MIP3660, GST-MIP3655 and GST-MIP0075) and the protein products were resolved by SDS-PAGE. Purple arrowheads, ~75 kDa IgM heavy chain; blue arrowheads, ~50–55 kDa IgG and IgA heavy chains; green arrowheads, ~65 kDa cleaved IgM heavy chain; red arrowheads, ~40 kDa cleaved IgG and IgA heavy chains; orange arrowheads, ~25 kDa IgM, IgA and IgG light chains.

As the recombinant GST-MIB proteins bound HRP-conjugated goat antibody in western blots, we hypothesised that the MIB proteins of *M. gallisepticum* may also interact with the IgGs of other mammals. Three bands were detected in binding assays using goat, sheep, rat, rabbit, horse, pig or tammar wallaby sera with all five MIB proteins, a ~75 kDa band suggestive of the mammalian IgM heavy chain, a ~50–55 kDa band suggestive of the IgG and IgA heavy chains and the ~25 kDa band indicative of the IgG, IgM and IgA light chain, in addition to the ~100 kDa GST-MIB protein (Figure 5). This indicated that all five MIB proteins in *M. gallisepticum* were able to bind mammalian IgG, IgM and IgA.

MIP cleavage assays showed that the GST-MIP3920 and GST-MIP3865 proteins that cleaved avian immunoglobulins were unable to cleave MIB-bound mammalian IgG, IgA or IgM (Figure 5). Only GST-MIP3660 was able to cleave MIB-bound mammalian IgG and IgA heavy chains (~50–55 kDa), producing a ~40 kDa cleaved fragment, and MIB-bound mammalian IgM heavy chains (~75 kDa), producing a ~65 kDa cleaved fragment. This cleavage was observed with goat, sheep, rat, rabbit, horse and pig immunoglobulins, but was not evident with MIB-bound immunoglobulins from tammar wallaby sera (Figure 5).

### Predicted cleavage site in IgY

**Figure 6:**
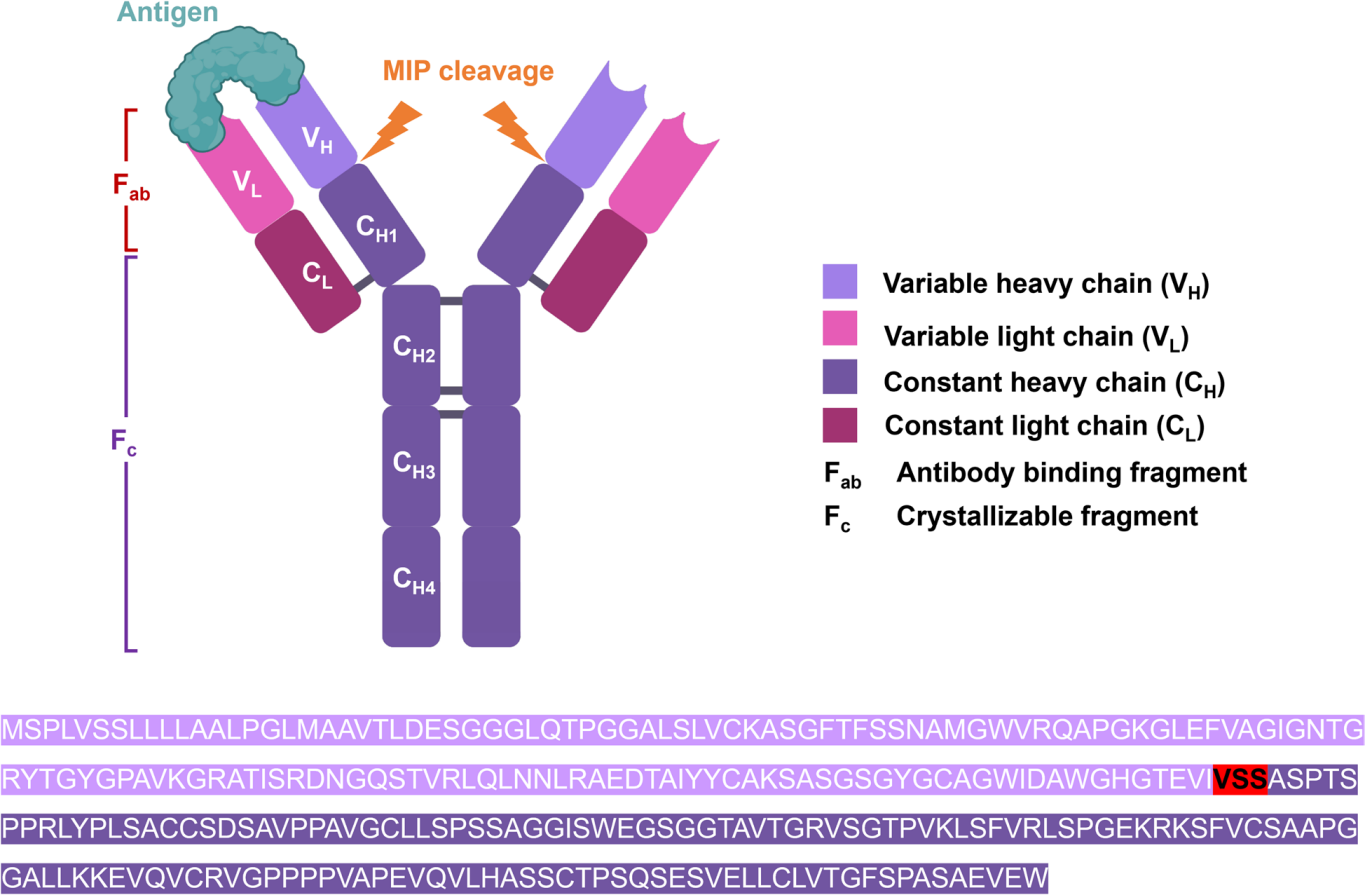
**Predicted cleavage site in avian IgY.** The MIP proteases are predicted to cleave the Fab fragment between the V_H_ domain (light purple) and the C_H1_ domain (dark purple) of the IgY heavy chain following the VSS amino acid sequence (highlighted in red). Image created using BioRender.com.

The molecular weight of the whole IgY binding with MIB proteins was ~180 kDa, comprising two IgY heavy chains (~65 kDa), each containing a variable V_H_ domain and four constant (C_H1–_C_H4_) domains, and two IgY light chains (~27 kDa), each with the variable V_L_ and constant C_L_ domains [40, 41] (Figs. 3 and 6). The cleaved IgY heavy chain was ~50 kDa (Figure 3B), suggesting a ~15 kDa fragment had been lost from the termini of the IgY heavy chains. Thus, the cleavage site was predicted to lie between the V_H_ and C_H1_ domains at the N-terminus or the C_H3_ and C_H4_ domains at the C-terminus of the IgY heavy chain (Figure 6). GST-MIP3660 was able to cleave mammalian IgG, resulting in a cleaved heavy chain of ~40 kDa (Figure 5). A protein sequence alignment of the IgY heavy chain sequences of chickens and ducks, and the IgG heavy chains of goats, sheep, rats, rabbits, horses and pigs, revealed a conserved putative cleavage site between the V_H_ and C_H1_ domains, immediately following the VSS sequence in the V_H_ domains (Additional file 16). This suggests that the MIPs of *M. gallisepticum* cleave chicken IgY at this conserved site, resulting in the irreversible separation of the Fab and Fc fragments (Figure 6).

### Distribution of, sequence similarity between, and possible recombination events between the MIB-MIP system genes of *M. gallisepticum* and other avian mycoplasmas

MIB-MIP gene homologues were identified in many other avian mycoplasmas, including *M. synoviae*, *M. imitans*, *M. tullyi*, *M. bradburyae*, *M. pullorum*, *M. gallinarum*, *M. meleagridis*, *M. gallinaceum, M. anatis*, *M. anseris*, *M. iners*, *M. gallopavonis*, *M. glycophilum* and *M. cloacale* (Table 2), but not in *M. iowae*, *M. lipofaciens* or *Ureaplasma gallorale*. In all species with homologues, the MIB-MIP genes were located close to the two genes encoding the two F_0_F_1_ ATP synthase subunits (Additional file 1).

**Table 2:** MIB-MIP homologues in other avian mycoplasmas.

Phylogenic group	Species	Known hosts	Pathogenicity	MIB homologues (gene locus tag/ORF)	MIP homologues (gene locus tag/ORF)
Pneumoniae	*M. imitans*	Chickens, turkeys, ducks, geese and partridges	Pathogenic	*P690_RS04465* *P690_RS04560* *P690_RS04555* *P690_RS0102830* *P690_RS0102825*	*P690_RS0102640* *P690_RS0102635*
	*M. tullyi*	Penguins	Pathogenic	*H3143_RS03375* *H3143_RS03370* *H3143_RS03365* *H3143_RS03360*	*H3143_RS03265* *H3143_RS03260*
	*M. bradburyae*	Seabirds	Pathogenic	*NMG68_RS03800* *NMG68_RS03890* *NMG68_RS03790* *NMG68_RS03785* *NMG68_RS03780* *NMG68_RS03775*	*NMG68_RS03665* *NMG68_RS03670*
	*M. iowae*	Chickens, turkeys	Pathogenic	None	None
	*U. gallorale*	Chickens, turkeys	Pathogenic	None	None
Hominis	*M. synoviae*	Chickens, turkeys	Pathogenic	*DEH79_02360* *DEH79_02405* *DEH79_00395* *DEH79_00390*	*DEH79_02365* DEH79_02410* *DEH79_02640*
	*M. meleagridis*	Chickens, turkeys	Pathogenic	*EXC33_RS02035*	*EXC33_RS02040*
	*M. gallinarum*	Chickens, turkeys	Non-pathogenic	*Q346_RS0101390*	*Q346_RS0102250* *Q346_RS04025*
	*M. pullorum*	Chickens, turkeys	Non-pathogenic	*BLA55_RS01610*	*BLA55_RS01655* *BLA55_RS01650*
	*M. anatis*	Ducks and geese	Pathogenic	*DP067_00625* *DP067_00620*	*DP067_02090*
	*M. anseris*	Ducks and geese	Pathogenic	*DP065_01310*	*DP065_01085* *DP065_01305*
	*M. cloacale*	Turkeys, ducks and geese	Pathogenic	*DK849_00535*	*DK849_00530* *DK849_00525*
	*M. gallopavonis*	Chickens, turkeys	Non-pathogenic	*NCTC10186_00734* *NCTC10186_00656*	*NCTC10186_00722*
	*M. glycophilum*	Chickens, turkeys, ducks and geese	Unknown	*NCTC10194_00200*	*NCTC10194_00208* *NCTC10194_00646*
	*M. iners*	Chickens, turkeys	Pathogenic	*T395_RS0102835*	*T395_RS0102840*
	*M. lipofaciens*	Chickens, turkeys, ducks and geese	Pathogenic	None	None

^*^Pseudogene.

**Figure 7:**
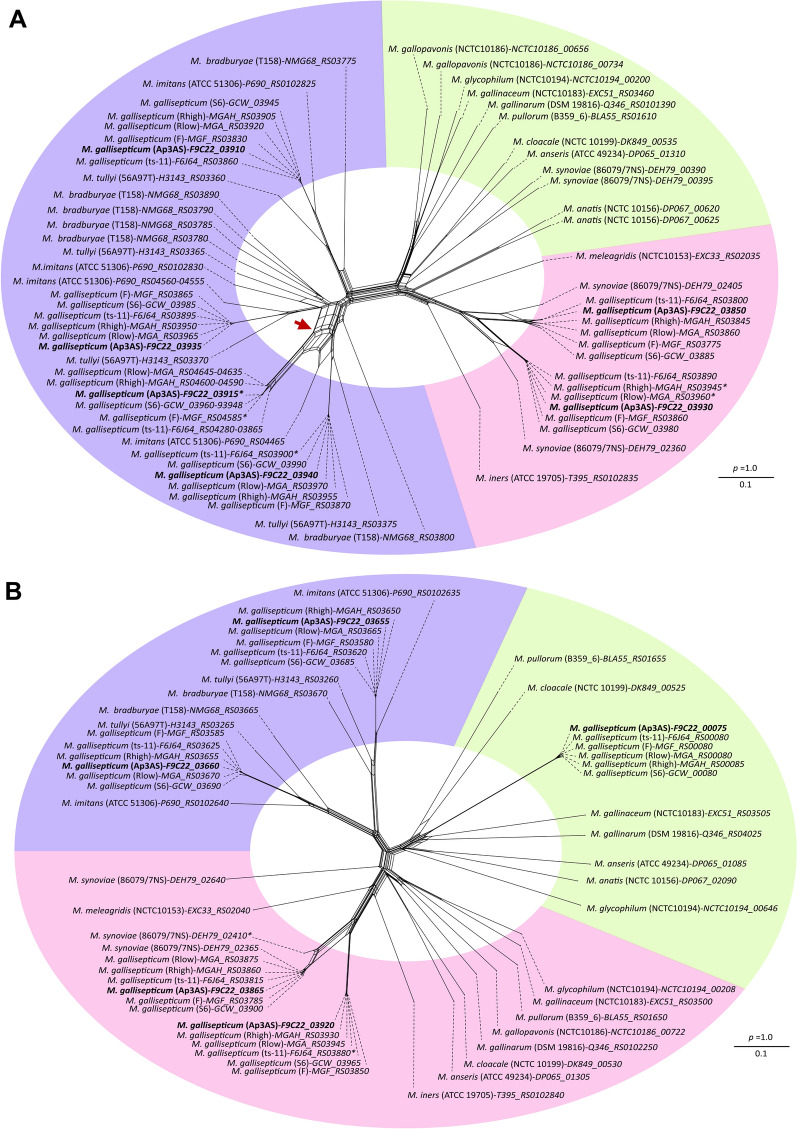
**Splits-decomposition phylogenetic network of MIB and MIP homologues of avian mycoplasmas.** Splits-decomposition networks were generated with aligned nucleotide sequences of putative MIB (**A**) and MIP (**B**) homologues of avian mycoplasmas, including multiple *M. gallisepticum* strains. The MIB and MIP homologues of avian mycoplasmas were distributed among three clusters colour coded as purple, pink and green based on the phylogenetic inference. The red arrowhead indicates the extensive reticulation network between the MIB homologues of *M. gallisepticum*, *M. imitans* and *M. tullyi*. The MIB-MIP homologues of *M. gallisepticum* strain Ap3AS are shown in bold. The parallel lines indicate a split. Pseudogenes are represented by an asterisk (*). Recombination analysis performed using the Pairwise Homoplasy Index (PHI) test indicated no significant recombination among MIB and MIP homologues (*p* = 1.0).

A splits-decomposition phylogenetic network distributed the MIB homologues from *M. gallisepticum* into two distinct clusters (Figure 7A). In the first cluster, the MIB homologues *F9C22_03910*, *03935*, *03940* and the pseudogene *F9C22_03915* grouped closely with *M. imitans* homologues, with which they shared 53–87% nucleotide sequence identity (Additional file 8) and 83–92% protein sequence similarity (74–85% identity) (Additional file 17). They were also related to homologues in *M. tullyi* (84–86% amino acid sequence similarity and 73–79% identity) and *M. bradburyae* (70–75% amino acid sequence similarity and 57–61% identity) (Additional file 17). The second cluster comprised the MIB homologues *F9C22_03930* and *F9C22_03850*, along with their homologues in other *M. gallisepticum* strains, and grouped with *DEH79_02360* and *DEH79_02405* of *M. synoviae* (Figure 7A). *F9C22_03930* and *DEH79_02360* shared 83% nucleotide sequence identity (Additional file 8) and 85% protein sequence similarity (77% identity) (Additional file 17), while *F9C22_03850* and *DEH79_02405* had 82% nucleotide sequence identity (Additional file 8) and 84% protein sequence similarity (78% identity) (Additional file 17). *F9C22_03930* and *F9C22_03850* were also distantly related to the single MIB homologue in *M. meleagridis* and *M. iners* (55–57% protein sequence similarity and 42–44% identity (Additional file 17)). The Pairwise Homoplasy Index (PHI) test in SpitsTree did not detect significant gene recombination (*p* = 1.0) between the MIB homologues of avian mycoplasmas. However, an extensive reticulation network suggestive of recombination was observed between the MIB homologues of *M. imitans*, *M. tullyi* and the related homologues in *M. gallisepticum* (Figure 7A). A separate PHI test performed with MIB homologues of just these species suggested significant gene recombination (*p* < 1.0) between those MIB homologues. This was further confirmed using RDP4 analysis, which predicted significant recombination (*p* < 0.05) between the MIB homologues of *M. gallisepticum* and *M. imitans*, mostly related to the *F9C22_03915* pseudogene and MIB homologues in *M. imitans*. It also predicted recombination events among the MIB homologues of other avian mycoplasmas, which were not detected by the PHI test (Additional file 18).

The MIP homologues of *M. gallisepticum* were distributed into three distinct clusters (Figure 7B). The MIP homologues *F9C22_03660* and *F9C22_03655* of *M. gallisepticum* were closely related to *P690_RS0102640* (73% nucleotide sequence identity, 81% amino acid sequence similarity and 73% amino acid sequence identity) and *P690_RS0102635* (86% nucleotide sequence identity, 91% amino acid sequence similarity and 85% amino acid sequence identity 85%) of *M. imitans* (Additional files 11 and 19). Both the MIP paralogues *F9C22_03920* and *F9C22_03865*, which had the highest amino acid sequence similarity, were closely related to *DEH79_02365* and the *DEH79_02410* pseudogene of *M. synoviae* (Figure 7B). *DEH79_02365* shared 97% nucleotide sequence identity and 95% protein sequence similarity (93% identity) with *F9C22_03865*, and 78% nucleotide sequence identity and 84% protein sequence similarity (72% identity) with *F9C22_03920* (Additional files 11 and 19). These two MIP homologues of *M. gallisepticum* were also related to the MIP homologue of *M. meleagridis* (55–59% nucleotide sequence identity, 61–66% amino acid sequence similarity and 44–47% amino acid sequence identity) (Additional files 11 and 19). *F9C22_00075* was not located within either of these clusters and was distantly related to all the other avian MIP homologues. No significant recombination was detected between the MIP homologues (*p* = 1.0) of the avian mycoplasmas using RDP4 or PHI test analysis.

### Possible horizontal gene transfer events between *M. gallisepticum* and other avian mycoplasmas

**Figure 8:**
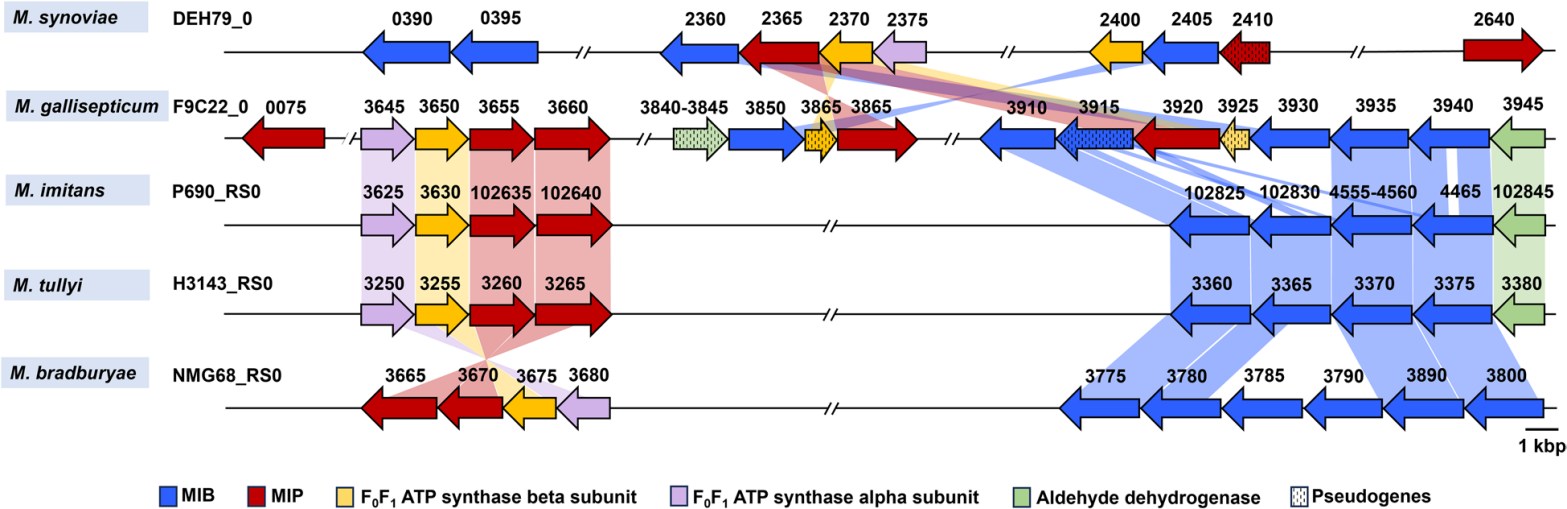
**Schematic representation of closely related MIB and MIP homologues and their associated gene regions among avian mycoplasmas.** The regions of the genome containing the putative MIB and MIP genes and the surrounding genes in *M. gallisepticum* strain Ap3AS, *M. synoviae* strain 86079/7NS, *M. imitans* strain ATCC 51306, *M. tullyi* strain 56A97T and *M. bradburyae* strain T158 are shown. Red arrows indicate putative MIP homologues; blue arrows, putative MIB homologues; yellow arrows, putative F_0_F_1_ ATP synthase subunit beta homologues; purple arrows, putative F_0_F_1_ ATP synthase subunit alpha homologues; green arrows, putative aldehyde dehydrogenase homologues. Homologous genes showing more than 70% nucleotide identity between each strain are linked by shaded areas representing the colours of each homologue, which indicate both homology and gene rearrangements such as inversions. Pseudogenes are indicated with vertical dashed hatching.

A Mauve analysis was performed using whole genome alignments to predict homologous MIB-MIP gene regions in the genomes of *M. gallisepticum* and other avian mycoplasmas. We found that the gene order in the MIB-MIP gene clusters in the *M. gallisepticum* genome was shared with *M. synoviae*, *M. imitans* and *M. tullyi* (Figure 8). The region encoding the *F9C22_03920* MIP homologue and the truncated *F9C22_03925* pseudogene was homologous to the *DEH79_02365* MIP homologue and part of the *DEH79_02370* F_0_F_1_ ATP synthase beta gene of *M. synoviae*, and, in the reverse orientation, these *M. synoviae* genes were also homologous to the *F9C22_03865* MIP homologue and the truncated *F9C22_03860* pseudogene of *M. gallisepticum*, suggesting a gene inversion. The MIB homologue *F9C22_03930* was homologous to *DEH79_02360* of *M. synoviae* and, in the reverse orientation, the *F9C22_03850* MIB homologue aligned with *DEH79_02405* of *M. synoviae* from a separate gene cluster. The flanking regions upstream (*F9C22_03910*–*F9C22_03915*) and downstream (*F9C22_03935*–*F9C22_03945*) of the *F9C22_03920*–*F9C22_03930* cluster were homologous to the putative MIB gene cluster in *M. imitans* and *M. tullyi*. The *F9C22_03915* MIB pseudogene contained a combination of conserved regions homologous to three MIB homologues of *M. imitans* (*P690_RS0102830*, *P690_RS04560*–*04555* and *P690_RS04465*). The gene region encompassing the *F9C22_03645*–*F9C22_03660* gene cluster was conserved across *M. gallisepticum*, *M. imitans* and *M. tullyi*, without any gene rearrangements, and was also conserved in *M. bradburyae*, though the entire region was inverted.

### MIP protein sequences surrounding the catalytic serine residue are conserved in different avian mycoplasmas

The protein sequence alignment of MIP homologues in avian mycoplasmas revealed a high level of conservation in the amino acid sequences in the vicinity of the catalytic serine residue, with two glycine (G) residues conserved across all sequences resulting in a G-SG catalytic site sequence (Additional file 20). Most homologues, including the three functional MIPs in *M. gallisepticum* strain Ap3AS (*F9C22_03920*, *F9C22_03865*, and *F9C22_03660*) had the sequence GGASG at the catalytic serine (S) residue (Additional file 20), identical to that found in the four functional MIPs in the ruminant pathogen *M. mycoides* ssp. *capri* (Additional file 3). Similar amino acid conservation was seen in the closely related homologues in *M. synoviae*, *M. imitans* and *M. tullyi* (Additional file 20). *F9C22_0075* encoded a YGASG sequence, while *F9C22_03655* and its homologues in *M. imitans and M. tullyi* encoded a PGGSG sequence, while *M. bradburyae* homologue encoded a GGGSG sequence. The GGASG sequence was also conserved in the MIP proteins of *M. meleagridis* and *M. iners*. The GASG sequence surrounding the catalytic serine was conserved in the majority of the avian mycoplasma MIP proteins (Additional file 20).

## Discussion

These studies have demonstrated that *M. gallisepticum* encodes multiple MIB-MIP proteins that can capture and cleave avian immunoglobulins. All five putative MIB proteins in *M. gallisepticum* are structurally similar to the IgG-blocking virulence protein of *M. mycoides* ssp. *capri* [20] and protein M of *M. genitalium* [42]. Functionally, these MIB proteins act as high-affinity IgY, IgM and IgA binding proteins*,* but have differing Ig-binding capacities, with the MIB protein encoded by *F9C22_03910* having the strongest binding capacity. These MIB proteins also have broad host specificity, binding to both avian and mammalian immunoglobulins. Of the five putative MIP proteins in *M. gallisepticum*, only three, encoded by *F9C22_03920, F9C22_03865* and *F9C22_03660,* function as active immunoglobulin endopeptidases, cleaving IgY, IgM and IgA bound to any of the five MIB proteins, although the capacity of these three functional MIP proteins to cleave immunoglobulins varies. The MIP proteins encoded by *F9C22_03920* and *F9C22_03865*, which share higher levels of amino acid sequence similarity, selectively cleaved avian immunoglobulins and had no activity against mammalian immunoglobulins, suggesting host adaptation. In contrast, the MIP protein encoded by *F9C22_03660*, which has a lower level of amino acid sequence similarity with these MIP proteins, cleaves mammalian and avian immunoglobulins. The broader binding specificity of the MIB proteins and the more selective specificity of the MIP-mediated cleavage indicates that antibody capture and proteolysis are distinct steps. The MIP-mediated cleavage may be affected by both the conformations of the different MIP proteins and structural differences between mammalian and avian antibodies [40], which together determine access to the V_H_-C_H1_ linker region.

The studies described here further revealed that a single IgY molecule can form a stable complex with MIBs, engaging with one or potentially two MIB proteins, most likely through multiple interactions with the light chain spanning the C_L_ and V_L_ domains, as previously observed with mammalian IgG [21]. The MIP proteases cleaved the MIB protein-bound IgY heavy chain between the V_H_-C_H1_ linker, releasing the V_H_ domains, but the cleaved IgY molecule remained tightly bound to the MIB, mirroring the IgG cleavage mechanism described for the MIB-MIP protein system in *M. mycoides* ssp. *capri* [20, 21]. As the V_H_ domain of the IgY heavy chain contains the three complementarity-determining regions, CDR1, CDR2 and CDR3, essential for antigen binding [40, 43] and for maintaining the structural stability of the antigen-binding site [44], its removal is likely to disrupt the formation of antigen–antibody complexes [5], undermining the host's ability to mount an effective immune response against infection with *M. gallisepticum* and the capacity of the humoral response to limit these infections.

Upon entering the host, *M. gallisepticum* is exposed to a humoral response, with the initial IgM response during the acute phase followed by IgA and IgY responses in the later chronic stages of infection [17, 45, 46]. These immunoglobulins, particularly IgY and IgA, which are abundant in body fluids and mucosal secretions such as respiratory secretions, play a crucial role in mucosal immunity [17, 46, 47]. Despite this, *M. gallisepticum* can colonize the respiratory tract, persist and disseminate systemically to distant sites [8, 10], underscoring the importance of the MIB-MIP system as a key immune evasion mechanism. This system may also facilitate transmission of *M. gallisepticum* by disrupting maternally derived IgM and IgA present in the egg white, as well as IgY in the egg yolk [47, 48], which are transferred via the oviduct and confer passive immunity to day-old chicks [49, 50]. Despite the presence of passively transferred maternal antibodies, *M. gallisepticum* can be transmitted from infected hens to embryos, surviving in embryos and vitelline membranes before subsequently infecting day-old chicks [8, 51], suggesting a potential involvement of the MIB-MIP system in overcoming maternal antibody defences, facilitating vertical transmission. As the MIB-MIP system of *M. gallisepticum* has antibody-binding and cleavage capacities comparable to those of the MIB-MIP proteins of mammalian mycoplasmas, it is likely it can also dissociate antigen–antibody complexes [21]. By releasing the bound antigens from immune complexes and irreversibly degrading the immunoglobulins, this system may further impair the host's ability to recognise antigens during subsequent infections, thereby subverting host immune responses [5, 21]. This may contribute to the establishment of the *M. gallisepticum* polymicrobial disease complex [7, 51], by interfering with humoral responses against other respiratory pathogens, allowing them to successfully colonise the respiratory tract and exacerbate disease initiated by *M. gallisepticum*.

Proteolytic cleavage by the MIP proteins of *M. gallisepticum* differs from that effected by the CysP protease, an IgY-specific enzyme found in *M. synoviae*, as this enzyme directly cleaves the IgY heavy chain without the need for an Ig-binding protein [52]. The MIP proteins in *M. gallisepticum* are unable to cleave IgY unless it is complexed with a MIB protein. This suggests that, as in *M. mycoides* ssp. *capri*, the MIB-MIP proteins of *M. gallisepticum* may act together to orient the immunoglobulin heavy chain and position the V_H_-C_H1_ cleavage site at the MIP catalytic site, enabling cleavage at a site that is otherwise inaccessible to the MIP protease [20, 21]. The three MIP proteins in *M. gallisepticum* share the same conserved GGASG motif, with a catalytic serine residue, seen in the MIP protein of *M. mycoides* ssp. *capri* [20], so it is possible that they also require interaction with the MIB-immunoglobulin complex to expose or activate the serine catalytic triad during cleavage. This conserved motif is also conserved in the putative MIP proteins encoded in the genomes of other avian mycoplasma species, so it is likely that these proteins are also able to cleave immunoglobulins. Although they contain a DUF31 domain and are annotated as putative serine proteases, the proteins encoded by *F9C22_03655* and *F9C22_00075* did not interact with avian or mammalian immunoglobulins, suggesting that their catalytic function may have been lost because of amino acid substitutions near the catalytic serine site. For instance, substitutions like the prolines either side of the motif in F9C22_03655 could affect the folding, stability and conformation of the protein [53–55], potentially disrupting the structural configuration required for catalytic activity. These changes may impair MIP activation or proteolysis, although further studies are needed to evaluate such effects. It is possible that *F9C22_03655* and *F9C22_00075* are remnants of once functional MIP protein genes that have lost their ability to cleave immunoglobulins. As the three functional MIP proteins can interact with all five MIB proteins, F9C22_03655 and F9C22_00075 might also serve alternative roles in *M. gallisepticum* that remain to be elucidated. The presence of multiple interacting paralogues across the *M. gallisepticum* genome reflects functional redundancy in the MIB-MIP system, which may enhance the adaptability of the immune evasion mechanisms of this organism [56]. However, the full role of these multiple interacting homologues is still unclear. Despite the high degree of conservation of the sequences of the MIB-MIP homologues, as well as their genomic arrangements, across *M. gallisepticum* strains, some strains, including S6, and the vaccines F and ts-11, contain frameshifted pseudogene variants that have likely lost function. The effects of these changes on virulence, immune evasion, and vaccine efficacy remain unknown and require further investigation.

As in the mammalian mycoplasmas, the MIB-MIP homologues of avian mycoplasmas are co-located with genes encoding the putative alpha and beta subunits of an F_0_F_1_ ATP synthase [21, 57]. Disruption of these ATP synthase subunits has recently been shown to impair the function of the MIB-MIP system in vivo in *M. mycoides* subsp. *capri* [57], and thus they are likely to play a similar role in the avian mycoplasma species.

The findings of the studies described here show that the MIB-MIP system is widely distributed among both pathogenic and non-pathogenic avian mycoplasmas that share similar avian hosts, predilection sites and transmission patterns [8, 58], consistent with previous findings in other mycoplasmas [20]. Several MIB-MIP homologues of *M. gallisepticum* are closely related to those in *M. imitans*, *M. tullyi* and *M. bradburyae*, which also belong to the pneumoniae phylogenetic cluster of mycoplasmas and share significant genome similarity [59–61]. Although *M. gallisepticum* and *M. synoviae* are distantly related and belong to two different phylogenetic groups, some *M. gallisepticum* MIB-MIP homologues are closely related to those in *M. synoviae*. This likely reflects horizontal gene transfer between these major pathogens, which has been reported previously for several other virulence-related loci [4, 52, 62], including the phase variable *vlhA* genes, which encode the major cell surface lipoproteins in both species, and are thought to play a critical role in immune evasion though antigenic variation [4, 63]. The scattered distribution of the MIB-MIP protein genes in the *M. gallisepticum* genome suggests multiple horizontal gene transfer events involving these homologues. Similarly, the MIB-MIP homologues of *M. meleagridis* and *M. iners* are also closely similar to those in *M. gallisepticum* and *M. synoviae*, suggesting further horizontal gene transfer events among these pathogens. Even non-pathogenic mycoplasmas, like *M. gallinarum*, *M. pullorum*, *M. gallinaceum* and *M. gallopavonis*, which are commonly isolated from birds concurrently with *M. gallisepticum* [58], have MIB-MIP homologues, suggesting the acquisition and retention of these homologues may provide an evolutionary advantage.

The MIB-MIP proteins of *M. gallisepticum* exhibit considerable amino acid sequence variation, likely driven by horizontal gene transfer from species like *M. synoviae* [4, 62] and subsequent recombination events, particularly between the MIB homologues. This variability might be an adaptive strategy to sustain effective MIB-MIP functionality in a maturing humoral immune response in which specific antibodies are generated against some of these proteins over the course of an infection.

This study primarily used in vitro approaches to characterise the MIB-MIP system of *M. gallisepticum*, and this may not fully capture the function of these proteins during infection in vivo. The expression patterns of the genes encoding the MIB-MIP paralogues across different stages of infection with *M. gallisepticum* and during transmission remain to be investigated. This limits our ability to determine whether these genes are differentially or adaptively expressed in response to host immune pressures. It would be interesting to investigate whether *M. gallisepticum* regulates MIB-MIP activity to adaptively evade specific host immune responses during transmission and over the course of infection in future studies.

## Conclusion

This study characterised the host-adaptation of the MIB-MIP immunoglobulin evasion system in *M. gallisepticum*, which comprises five high-affinity MIB immunoglobulin-binding proteins and three functional MIP proteases, which selectively cleave avian immunoglobulins bound to any of the five MIB proteins. This coordinated mechanism enables *M. gallisepticum* to undermine the host immune response, facilitating persistent infection and transmission. The widespread presence of the MIB-MIP system among avian mycoplasmas, together with its broad functional specificity in *M. gallisepticum* and detection of horizontal gene transfer and recombination signatures within these loci, suggests that MIB-MIP systems have undergone repeated evolutionary reshaping, likely facilitating host adaptation and persistence across avian hosts. Together, these findings link immune evasion, host specificity and genome plasticity in *M. gallisepticum* and related mycoplasmas. This advances our understanding of host–pathogen interactions in avian mycoplasmas and may inform future approaches to control mycoplasma infections in poultry.

## Supplementary Information

**Additional file 1. MIB-MIP homologues and associated ATPase homologues in avian mycoplasma species.**

**Additional file 2. Internal primers used to confirm MIB-MIP insert sequences.**

**Additional file 3. Conserved catalytic serine site in *M. gallisepticum* MIP homologues and functional MIP homologues of *M. mycoides* ssp. *capri*. **The amino acid sequences of the putative MIP proteins of *M. gallisepticum* strain Ap3AS and *M. mycoides* ssp. *capri *strain GM12 were aligned using EMBL-EBI Clustal Omega Multiple Sequence Alignment and processed using ESPript 3.0 to generate the graphical alignment. The conserved catalytic serine site is indicated by the green bar. Gaps are indicated by dots “.”, blue boxes indicate conserved regions, identical residues conserved across the sequences are shaded red, and the residues that share similar properties are indicated by red letters.

**Additional file 4. Protein sequence similarities and identities between MIP homologues of *M. gallisepticum *Ap3AS and *M. mycoides* ssp. *capri *GM12.**

**Additional file 5. Arrangement of putative MIB-MIP homologues in their gene clusters in different strains of *M. gallisepticum*. **Genomic regions encoding the putative MIBs, MIPs and surrounding genes were extracted from the complete genome sequences of *M. gallisepticum* strains Ap3AS, R_low_, R_high_, S6, F, and ts-11, and aligned using the progressive Mauve algorithm to identify homologous genes and gene rearrangements. Putative MIB homologues are indicated by blue arrows and MIP homologues by red arrows; putative F_0_F_1_ ATP synthase beta subunit by yellow arrows and alpha subunits by purple arrows; and putative aldehyde dehydrogenase homologues by green arrows. Pseudogenes are indicated with dashed arrowheads. Colinear homologues across the strains are shaded using the respective colours of the putative homologous genes.

**Additional file 6. Protein sequence similarities and identities between MIB homologues of *M. gallisepticum *Ap3AS and those of other strains of *M. gallisepticum*.**

**Additional file 7. Protein sequence similarities and identities between MIP homologues of *M. gallisepticum* Ap3AS and other strains of *M. gallisepticum*.**

**Additional file 8. Percentage pairwise nucleotide identities of MIB homologues.**

**Additional file 9. Protein sequence similarities and identities between MIB homologues of *M. gallisepticum *Ap3AS.**

**Additional file 10. Amino acid sequence similarities of putative MIB proteins. **The amino acid sequences of the putative MIB proteins were aligned to identify conserved amino acid residues. Gaps are indicated by dots “.”, residues that share similar properties by red letters, identical residues conserved across the sequences are shaded red, and conserved regions are indicated by blue boxes.

**Additional file 11. Percentage pairwise nucleotide identities of MIP homologues.**

**Additional file 12. Percentage sequence similarities and identities between MIP homologues of *M. gallisepticum *Ap3AS.**

**Additional file 13. Amino acid sequence similarities of putative MIP proteins. **The amino acid sequences of the putative MIB proteins were aligned to identify conserved amino acid residues. The green bar indicates the conserved catalytic serine site. Gaps are indicated by dots “.”, residues that share similar properties by red letters, identical residues conserved across the sequences are shaded red, and conserved regions are indicated by blue boxes.

**Additional file 14. Interaction of the putative MIB-MIP proteins of *M. gallisepticum *with immunoglobulins of different avian species. **Purified recombinant GST-MIB proteins (only GST-MIB3910 is shown as representative of the observation for all five MIB proteins) were incubated with chicken, turkey, pheasant or duck sera. Affinity purified MIB-immunoglobulin complexes were then incubated with purified recombinant GST-MIP proteins (GST-MIP3920, GST-MIP3865, GST-MIP3660, GST-MIP3655 and GST-MIP0075) and protein products were resolved by SDS-PAGE. Purple arrowheads indicate the ~75 kDa IgM and IgA heavy chains; blue arrowheads the ~65 kDa IgY heavy chain; green arrowheads the ~60 kDa cleaved IgM and IgA heavy chains; red arrowheads the ~55 kDa cleaved IgY heavy chain; and orange arrowheads the ~25 kDa IgM, IgA and IgY light chains.

**Additional file 15. Interaction of MIP proteins with unbound purified chicken IgY. **Purified recombinant proteins GST-MIP3920, GST-MIP3865, GST-MIP3660, GST-MIP3655 and GST-MIP0075 were incubated directly with purified chicken IgY and the products were separated by SDS-PAGE. A western blot was probed with anti-chicken IgY Fc HRP-conjugated antibody to identify the IgY heavy chain and detect any cleavage. Blue arrowheads, IgY heavy chain; orange arrowhead, IgY light chain.

**Additional file 16. Predicted cleavage site in the V**_**H**_** domain of immunoglobulins from different species. **Alignment of the amino acid sequences of the V_H_ domain of IgY from chicken (*Gallus gallus*) and ducks (*Anas platyrhynchos*); and IgG of goats (*Capra hircus*), sheep (*Ovis aries*), rats (*Rattus norvegicus*), rabbits (*Oryctolagus caniculus*), horses (*Equus caballus*) and pigs (*Sus domesticus*). The conserved VSS cleavage site at the carboxyl end of the V_H_ domain is present in all the immunoglobulin sequences and is indicated by the green bar underneath. Gaps are indicated by dots “.”, residues that share similar properties are indicated with red letters, identical residues conserved across the sequences are shaded red, and the conserved regions are indicated with blue boxes.

**Additional file 17. Protein sequence similarities and identities between MIB homologues of *M. gallisepticum *Ap3AS and those of other avian mycoplasmas.**

**Additional file 18. Summary of RDP4 recombination analysis for MIB homologues.**

**Additional file 19. Protein homology between MIP homologues in *M. gallisepticum* Ap3AS and other avian mycoplasmas.**

**Additional file 20. Conserved catalytic serine site in avian mycoplasma MIP homologues.** Alignment of amino acid sequences of the putative MIP proteins of avian mycoplasmas surrounding the conserved catalytic serine site (indicated by the green bar). Gaps are indicated by dots “.”, conserved regions are indicated by blue boxes, identical residues conserved across the sequences are shaded red, and the residues that share similar properties are represented by red letters.

## Data Availability

All the data generated or analysed during this study are included in this article and its additional files.
