# Supplementary material for: Host specificity and horizontal gene transfer in the MIB-MIP immunoglobulin evasion system in Mycoplasma gallisepticum
Source: Vet Res. 2026 Aug 11;57:145. doi: 10.1186/s13567-026-01779-x (PMC13459921; doi:10.1186/s13567-026-01779-x)
Supplement: Supplementary file 1 [file 13567_2026_1779_MOESM12_ESM.docx]

**Additional file 12. Percentage sequence similarities and identities between MIP homologues of *M. gallisepticum* Ap3AS**

|  | ***F9C22_03920*** | ***F9C22_03865*** | ***F9C22_03660*** | ***F9C22_03655*** | ***F9C22_00075*** |
| --- | --- | --- | --- | --- | --- |
| ***F9C22_03920*** | 100 (100) | 84 (72) | 40 (27) | 37 (23) | 31 (19) |
| ***F9C22_03865*** | 84 (72) | 100 (100) | 39 (25) | 34 (22) | 32 (19) |
| ***F9C22_03660*** | 40 (27) | 39 (25) | 100 (100) | 47 (33) | 31 (20) |
| ***F9C22_03655*** | 37 (23) | 34 (22) | 47 (33) | 100 (100) | 29 (17) |
| ***F9C22_00075*** | 31 (19) | 32 (19) | 31 (20) | 29 (17) | 100 (100) |

Percentage sequence similarities and identities (in brackets) were calculated based on a pairwise Needleman-Wunsch global alignment of protein sequences.
