# Supplementary material for: Host specificity and horizontal gene transfer in the MIB-MIP immunoglobulin evasion system in Mycoplasma gallisepticum
Source: Vet Res. 2026 Aug 11;57:145. doi: 10.1186/s13567-026-01779-x (PMC13459921; doi:10.1186/s13567-026-01779-x)
Supplement: Supplementary file 2 [file 13567_2026_1779_MOESM17_ESM.docx]

**Additional file 17. Protein sequence similarities and identities between MIB homologues of *M. gallisepticum* Ap3AS and those of other avian mycoplasmas**

| **Species** | **Gene locus tag** | ***M. gallisepticum* strain Ap3AS** | | | | |
| --- | --- | --- | --- | --- | --- | --- |
|  |  | ***F9C22_03940*** | ***F9C22_03935*** | ***F9C22_03930*** | ***F9C22_03910*** | ***F9C22_03850*** |
| ***M. imitans* ATCC51306** | ***P690_RS04465*** | 86 (76) | 68 (52) | 45 (27) | 61 (47) | 49 (33) |
|  | ***P690_ RS04455-RS04460*** | 66 (52) | 83 (74) | 46 (29) | 61 (46) | 45 (27) |
|  | ***P690_RS0102830*** | 70 (54) | 77 (65) | 47 (31) | 65 (48) | 45 (28) |
|  | ***P690_RS010282*** | 63 (48) | 64 (48) | 48 (21) | 92 (85) | 47 (28) |
| ***M. tullyi* 56A97T** | ***H3143_RS03375*** | 88 (79) | 68 (53) | 45 (28) | 63 (48) | 47 (31) |
|  | ***H3143_RS03370*** | 66 (51) | 84 (73) | 48 (30) | 62 (47) | 49 (29) |
|  | ***H3143_RS03365*** | 69 (54) | 76 (62) | 48 (31) | 65 (48) | 46 (29) |
|  | ***H3143_RS03360*** | 65 (51) | 63 (49) | 45 (29) | 86 (75) | 46 (30) |
| ***M. bradburyae* T158** | ***NMG68_RS03800*** | 72 (60) | 65 (49) | 45 (28) | 62 (48) | 46 (29) |
|  | ***NMG68_RS03890*** | 69 (54) | 75 (57) | 48 (28) | 65 (48) | 45 (28) |
|  | ***NMG68_RS03790*** | 68 (52) | 72 (57) | 47 (28) | 62 (47) | 45 (27) |
|  | ***NMG68_RS03785*** | 65 (51) | 70 (54) | 46 (28) | 63 (47) | 44 (27) |
|  | ***NMG68_RS03780*** | 69 (55) | 75 (61) | 46 (28) | 66 (50) | 45 (28) |
|  | ***NMG68_RS03775*** | 60 (46) | 61 (43) | 43 (26) | 75 (60) | 45 (26) |
| ***M. synoviae* 86079/7NS** | ***DEH79_02360*** | 44 (26) | 44 (27) | 85 (77) | 43 (27) | 75 (62) |
|  | ***DEH79_02405*** | 41 (26) | 44 (26) | 74 (63) | 43 (27) | 84 (78) |
|  | ***DEH79_00395*** | 42 (23) | 41 (21) | 50 (32) | 42 (25) | 47 (29) |
|  | ***DEH79_00390*** | 41 (23) | 39 (21) | 49 (30) | 40 (24) | 48 (30) |
| ***M. meleagridis* NCTC10153** | ***EXC33_RS02035*** | 41 (24) | 38 (24) | 55 (42) | 41 (26) | 55 (43) |
| ***M. iners***  **ATCC19705** | ***T395_RS0102835*** | 41 (27) | 40 (26) | 57 (43) | 42 (26) | 57 (44) |
| ***M. gallinarum* DSM19816** | ***Q346_RS0101390*** | 26 (15) | 27 (15) | 28 (17) | 26 (16) | 28 (17) |
| ***M. pullorum* B359_6** | ***BLA55_RS01610*** | 27 (16) | 28 (16) | 31 (18) | 29 (17) | 31 (19) |
| ***M. gallinaceum* NCTC10183** | ***EXC51_RS03460*** | 29 (16) | 30 (17) | 31 (19) | 30 (17) | 32 (20) |
| ***M. gallopavonis* NCTC10186** | ***NCTC10186_00734*** | 28 (15) | 29 (17) | 32 (19) | 30 (17) | 30 (18) |
|  | ***NCTC10186_00656*** | 27 (15) | 29 (17) | 31 (20) | 29 (17) | 31 (20) |
| ***M. glycophilum* NCTC10194** | ***NCTC10194_00200*** | 27 (15) | 31 (17) | 30 (18) | 29 (17) | 32 (20) |
| ***M. anatis* NCTC10156** | ***DP067_00625*** | 23 (15) | 23 (15) | 29 (19) | 25 (16) | 29 (19) |
|  | ***DP067_00620*** | 24 (15) | 24 (15) | 27 (18) | 24 (16) | 28 (20) |
| ***M. anseris* ATCC49234** | ***DP065_01310*** | 29 (17) | 29 (18) | 34 (24) | 29 (18) | 35 (23) |
| ***M. cloacale* NCTC10199** | ***DK849_00535*** | 27 (17) | 28 (16) | 33 (22) | 27 (17) | 35 (23) |

Percentage sequence similarities and identities (in brackets) were calculated based on a pairwise Needleman-Wunsch global alignment of protein sequences.
