# Supplementary material for: Host specificity and horizontal gene transfer in the MIB-MIP immunoglobulin evasion system in Mycoplasma gallisepticum
Source: Vet Res. 2026 Aug 11;57:145. doi: 10.1186/s13567-026-01779-x (PMC13459921; doi:10.1186/s13567-026-01779-x)
Supplement: Supplementary file 3 [file 13567_2026_1779_MOESM19_ESM.docx]

**Additional file 19. Protein homology between MIP homologues in *M. gallisepticum* Ap3AS and other avian mycoplasmas**

| **Species** | **Gene locus tag** | ***M. gallisepticum* strain Ap3AS** | | | | |
| --- | --- | --- | --- | --- | --- | --- |
|  |  | ***F9C22_03920*** | ***F9C22_03865*** | ***F9C22_03660*** | ***F9C22_03655*** | ***F9C22_00075*** |
| ***M. synoviae* 86079/7NS** | ***DEH79_02365*** | 84 (72) | 95 (93) | 39 (25) | 35 (23) | 31 (20) |
|  | ***DEH79_02640*** | 47 (33) | 48 (32) | 39 (25) | 36 (22) | 32 (20) |
| ***M. imitans* ATCC51306** | ***P690_RS0102640*** | 37 (24) | 37 (24) | 81 (73) | 46 (33) | 33 (19) |
|  | ***P690_RS0102635*** | 38 (23) | 35 (23) | 45 (33) | 91 (85) | 32 (18) |
| ***M. tullyi* 56A97T** | ***H3143_RS03265*** | 39 (25) | 39 (25) | 83 (73) | 46 (31) | 33 (20) |
|  | ***H3143_RS03260*** | 36 (23) | 35 (22) | 46 (33) | 84 (73) | 31 (19) |
| ***M. bradburyae* T158** | ***NMG68_RS03665*** | 39 (25) | 38 (24) | 73 (61) | 47 (31) | 31 (17) |
|  | ***NMG68_RS03670*** | 37 (22) | 36 (23) | 45 (32) | 79 (69) | 32 (18) |
| ***M. meleagridis* NCTC10153** | ***EXC33_RS02040*** | 61 (44) | 64 (47) | 40 (26) | 35 (24) | 33 (20) |
| ***M. gallinarum* DSM19816** | ***Q346_RS04025*** | 34 (20) | 33 (20) | 32 (18) | 32 (19) | 45 (29) |
|  | ***Q346_RS0102250*** | 51 (37) | 51 (37) | 41 (25) | 38 (25) | 32 (19) |
| ***M. pullorum* B359_6** | ***BLA55_RS01655*** | 34 (21) | 32 (20) | 33 (20) | 29 (18) | 44 (30) |
|  | ***BLA55_RS01650*** | 47 (31) | 47 (31) | 36 (23) | 37 (23) | 32 (19) |
| ***M. gallinaceum* NCTC10183** | ***EXC51_RS03505*** | 34 (19) | 35 (20) | 31 (19) | 30 (18) | 44 (28) |
|  | ***EXC51_RS03500*** | 47 (33) | 50 (34) | 40 (26) | 38 (25) | 34 (20) |
| ***M. anatis* NCTC10156** | ***DP067_02090*** | 27 (16) | 29 (17) | 33 (20) | 33 (21) | 29 (15) |
| ***M. anseris* ATCC49234** | ***DP065_01085*** | 30 (18) | 29 (16) | 26 (15) | 25 (16) | 28 (15) |
|  | ***DP065_01305*** | 48 (32) | 49 (32) | 39 (26) | 37 (24) | 35 (21) |
| ***M. cloacale* NCTC10199** | ***DK849_00530*** | 49 (34) | 51 (33) | 40 (25) | 37 (23) | 35 (20) |
|  | ***DK849_00525*** | 31 (21) | 32 (21) | 34 (22) | 35 (20) | 45 (32) |
| ***M. gallopavonis* NCTC10186** | ***NCTC10186_00722*** | 52 (36) | 51 (35) | 41 (25) | 39 (25) | 30 (17) |
| ***M. glycophilum* NCTC10194** | ***NCTC10194_00208*** | 50 (34) | 50 (35) | 39 (25) | 41 (26) | 31 (19) |
|  | ***NCTC10194_00646*** | 34 (20) | 32 (19) | 33 (18) | 33 (20) | 33 (21) |

Percentage sequence similarities and identities (in brackets) were calculated based on a pairwise Needleman-Wunsch global alignment of protein sequences.
