# Supplementary material for: Host specificity and horizontal gene transfer in the MIB-MIP immunoglobulin evasion system in Mycoplasma gallisepticum
Source: Vet Res. 2026 Aug 11;57:145. doi: 10.1186/s13567-026-01779-x (PMC13459921; doi:10.1186/s13567-026-01779-x)
Supplement: Supplementary file 4 [file 13567_2026_1779_MOESM2_ESM.docx]

**Additional file 2. Internal primers used to confirm MIB-MIP insert sequences.**

| **Gene locus tag** | **Oligonucleotide** | **Sequence (5′-3′)** |
| --- | --- | --- |
| **MIB homologues** | | |
| *F9C22_03940* | - | No internal primers required |
| *F9C22_03935* | - | No internal primers required |
| *F9C22_03930* | - | No internal primers required |
| *F9C22_03910* | CDS28fwd1 | GGTGCTGTAACTGCAACAGG |
| *F9C22_03850* | CDS40fwd1 | GCA AATGCTATTGGCTGGCC |
| **MIP homologues** | | |
| *F9C22_03920* | CDS24fwd1 | AGCAGAAGCACAACGTTATCA |
| *F9C22_03865* | CDS36fwd1 | TTAGCAACTAACGCTCACGTA |
| *F9C22_03660* | - | No internal primers required |
| *F9C22_03655* | - | No internal primers required |
| *F9C22_00075* | - | No internal primers required |
