# Supplementary material for: Host specificity and horizontal gene transfer in the MIB-MIP immunoglobulin evasion system in Mycoplasma gallisepticum
Source: Vet Res. 2026 Aug 11;57:145. doi: 10.1186/s13567-026-01779-x (PMC13459921; doi:10.1186/s13567-026-01779-x)
Supplement: Supplementary file 5 [file 13567_2026_1779_MOESM4_ESM.docx]

**Additional file 4. Protein sequence similarities and identities between MIP homologues of *M. gallisepticum* Ap3AS and *M. mycoides* ssp. *capri* GM12**

|  |  | ***M. gallisepticum* Ap3AS** | | | | |
| --- | --- | --- | --- | --- | --- | --- |
|  |  | ***F9C22_03920*** | ***F9C22_03865*** | ***F9C22_03660*** | ***F9C22_03655*** | ***F922_00075*** |
| ***M. mycoides* ssp. *capri*** | ***MMCAP2_RS02915*** | 41 (28) | 40 (25) | 36 (23) | 33 (21) | 31 (18) |
|  | ***MMCAP2_RS05110*** | 39 (25) | 40 (25) | 37 (24) | 35 (23) | 33 (18) |
|  | ***MMCAP2_RS02895*** | 43 (26) | 42 (26) | 36 (24) | 35 (21) | 33 (19) |
|  | ***MMCAP2_RS02885*** | 40 (24) | 39 (25) | 37 (23) | 35 (22) | 33 (20) |

Percentage similarities and identities (in brackets) were calculated based on a pairwise Needleman-Wunsch global alignment of protein sequences.
