# Supplementary material for: Host specificity and horizontal gene transfer in the MIB-MIP immunoglobulin evasion system in Mycoplasma gallisepticum
Source: Vet Res. 2026 Aug 11;57:145. doi: 10.1186/s13567-026-01779-x (PMC13459921; doi:10.1186/s13567-026-01779-x)
Supplement: Supplementary file 6 [file 13567_2026_1779_MOESM6_ESM.docx]

**Additional file 6. Protein sequence similarities and identities between MIB homologues of *M. gallisepticum* Ap3AS and those of other strains of *M. gallisepticum***

| **Strain** | | **Gene locus tag** | **Ap3AS** | | | | |
| --- | --- | --- | --- | --- | --- | --- | --- |
|  |  |  | ***F9C22_03940*** | ***F9C22_03935*** | ***F9C22_03930*** | ***F9C22_03910*** | ***F9C22_03850*** |
| **R_low_** | ***MGA_RS03970*** | | 99 (99) | 67 (54) | 45 (28) | 62 (48) | 48 (31) |
|  | ***MGA_RS03965*** | | 67 (53) | 99 (99) | 45 (29) | 64 (48) | 46 (29) |
|  | ***MGA_RS03920*** | | 62 (48) | 64 (49) | 47 (29) | 98 (98) | 44 (29) |
|  | ***MGA_RS03860*** | | 48 (31) | 47 (28) | 83 (73) | 46 (29) | 96 (95) |
| **R_high_** | ***MGAH_RS03955*** | | 99 (99) | 67 (54) | 45 (28) | 62 (48) | 48 (31) |
|  | ***MGAH_RS03950*** | | 67 (53) | 99 (99) | 45 (29) | 64 (48) | 46 (29) |
|  | ***MGAH_RS03905*** | | 62 (47) | 64 (49) | 47 (29) | 98 (98) | 44 (29) |
|  | ***MGAH_RS03845*** | | 48 (31) | 47 (28) | 83 (73) | 46 (29) | 96 (95) |
| **S6** | ***GCW_03990*** | | 99 (99) | 67 (54) | 47 (29) | 62 (47) | 48 (31) |
|  | ***GCW_03985*** | | 67 (53) | 98 (98) | 45 (29) | 64 (48) | 45 (29) |
|  | ***GCW_03980*** | | 45 (28) | 46 (30) | 98 (98) | 46 (28) | 82 (71) |
|  | ***GCW_03945*** | | 62 (47) | 64 (49) | 47 (29) | 99 (99) | 46 (30) |
|  | ***GCW_03885*** | | 48 (31) | 47 (28) | 82 (72) | 46 (29) | 95 (92) |
| **F** | ***MGF_RS03870*** | | 98 (98) | 67 (53) | 46 (28) | 62 (47) | 48 (32) |
|  | ***MGF_RS03865*** | | 67 (54) | 99 (98) | 45 (29) | 64 (48) | 46 (29) |
|  | ***MGF_RS03860*** | | 45 (28) | 46 (30) | 98 (98) | 46 (28) | 82 (71) |
|  | ***MGF_RS03830*** | | 62 (47) | 64 (49) | 47 (29) | 98 (98) | 45 (29) |
|  | ***MGF_RS03775*** | | 48 (31) | 47 (28) | 83 (72) | 46 (29) | 95 (92) |
| **ts-11** | ***F6J64_RS03895*** | | 85 (83) | 60 (48) | 43 (27) | 57 (42) | 46 (30) |
|  | ***F6J64_RS03890*** | | 46 (29) | 45 (29) | 99 (99) | 46 (28) | 82 (71) |
|  | ***F6J64_RS03860*** | | 63 (48) | 64 (49) | 47 (30) | 98 (97) | 46 (30) |
|  | ***F6J64_RS03800*** | | 48 (31) | 48 (29 | 82 (71) | 46 (27) | 99 (99) |

Percentage sequence similarities and identities (in brackets) were calculated based on a pairwise Needleman-Wunsch global alignment of protein sequences.
