# Supplementary material for: Host specificity and horizontal gene transfer in the MIB-MIP immunoglobulin evasion system in Mycoplasma gallisepticum
Source: Vet Res. 2026 Aug 11;57:145. doi: 10.1186/s13567-026-01779-x (PMC13459921; doi:10.1186/s13567-026-01779-x)
Supplement: Supplementary file 7 [file 13567_2026_1779_MOESM7_ESM.docx]

**Additional file 7. Protein sequence similarities and identities between MIP homologues of *M. gallisepticum* Ap3AS and other strains of *M. gallisepticum***

| **Strain and gene locus tag** | | **Ap3AS** | | | | |
| --- | --- | --- | --- | --- | --- | --- |
|  |  | ***F9C22_03920*** | ***F9C22_03865*** | ***F9C22_03660*** | ***F9C22_03655*** | ***F9C22_00075*** |
| **R_low_** | ***MGA_RS03945*** | 99 (99) | 84 (72) | 40 (26) | 37 (23) | 31 (19) |
|  | ***MGA_RS03875*** | 84 (72) | 98 (97) | 40 (26) | 34 (21) | 32 (19) |
|  | ***MGA_RS03670*** | 40 (27) | 39 (25) | 99 (99) | 47 (33) | 32 (20) |
|  | ***MGA_RS03665*** | 38 (24) | 34 (22) | 47 (33) | 99 (99) | 29 (18) |
|  | ***MGA_RS00080*** | 33 (20) | 33 (19) | 32 (20) | 30 (19) | 98 (97) |
| **R_high_** | ***MGAH_RS03930*** | 99 (99) | 84 (72) | 40 (26) | 37 (23) | 31 (19) |
|  | ***MGAH_RS03860*** | 84 (72) | 98 (97) | 40 (26) | 34 (21) | 32 (19) |
|  | ***MGAH_RS03655*** | 40 (27) | 39 (25) | 99 (99) | 47 (33) | 32 (20) |
|  | ***MGAH_RS03650*** | 38 (24) | 34 (22) | 47 (33) | 99 (99) | 29 (18) |
|  | ***MGAH_RS00085*** | 33 (20) | 33 (19) | 32 (20) | 30 (19) | 98 (97) |
| **S6** | ***GCW_03965*** | 98 (98) | 83 (72) | 40 (26) | 37 (23) | 31 (19) |
|  | ***GCW_03900*** | 83 (72) | 98 (97) | 38 (25) | 34 (22) | 33 (19) |
|  | ***GCW_03690*** | 39 (27) | 39 (26) | 98 (97) | 47 (33) | 31 (20) |
|  | ***GCW_03685*** | 38 (24) | 34 (23) | 47 (33) | 99 (98) | 30 (19) |
|  | ***GCW_00080*** | 32 (19) | 33 919) | 32 920) | 29 (17) | 97 (96) |
| **F** | ***MGF_RS03850*** | 98 (98) | 83 (72) | 40 (26) | 37 (23) | 30 (19) |
|  | ***MGF_RS03785*** | 84 (72) | 98 (97) | 39 (25) | 34 (22) | 33 (19) |
|  | ***MGF_RS03585*** | 40 (27) | 39 (25) | 99 (98) | 47 (33) | 32 (20) |
|  | ***MGF_RS03580*** | 38 (24) | 34 (23) | 47 (33) | 99 (99) | 30 (19) |
|  | ***MGF_RS00080*** | 31 (19) | 33 (20) | 31 (20) | 29 (17) | 99 (99) |
| **ts-11** | ***F6J64_RS03815*** | 84 (72) | 97 (96) | 39 (26) | 34 (22) | 30 (19) |
|  | ***F6J64_RS03625*** | 40 (27) | 39 (25) | 98 (99) | 47 (33) | 33 (20) |
|  | ***F6J64_RS03620*** | 38 (24) | 35 (23) | 47 (33) | 99 (99) | 30 (18) |
|  | ***F6J64_RS00080*** | 31 (19) | 33 (19) | 32 (20) | 29 (17) | 99 (98) |

Percentage sequence similarities and identities (in brackets) were calculated based on a pairwise Needleman-Wunsch global alignment of protein sequences.
