# Supplementary material for: Host specificity and horizontal gene transfer in the MIB-MIP immunoglobulin evasion system in Mycoplasma gallisepticum
Source: Vet Res. 2026 Aug 11;57:145. doi: 10.1186/s13567-026-01779-x (PMC13459921; doi:10.1186/s13567-026-01779-x)
Supplement: Supplementary file 8 [file 13567_2026_1779_MOESM9_ESM.docx]

**Additional file 9. Protein sequence similarities and identities between MIB homologues of *M. gallisepticum* Ap3AS**

|  | ***F9C22_03940*** | ***F9C22_03935*** | ***F9C22_03930*** | ***F9C22_03910*** | ***F9C22_03850*** |
| --- | --- | --- | --- | --- | --- |
| ***F9C22_03940*** | 100 (100) | 67 (54) | 45 (28) | 62 (47) | 48 (32) |
| ***F9C22_03935*** | 67 (54) | 100 (100) | 45 (29) | 64 (48) | 47 (29) |
| ***F9C22_03930*** | 45 (28) | 45 (29) | 100 (100) | 47 (29) | 82 (71) |
| ***F9C22_03910*** | 62 (47) | 64 (48) | 47 (29) | 100 (100) | 47 (30) |
| ***F9C22_03850*** | 48 (32) | 47 (29) | 82 (71) | 47 (30) | 100 (100) |

Percentage sequence similarities and identities (in brackets) were calculated based on a pairwise Needleman-Wunsch global alignment of protein sequences.
